# A Qualitative Serial Analysis of Drawings by Thirteen-to Fifteen-Year-Old Adolescents in Sweden About the First Wave of the Covid-19 Pandemic

**DOI:** 10.1177/10497323221101978

**Published:** 2022-05-21

**Authors:** Carol Tishelman, Johanna L. Degen, Sofía Weiss Goitiandía, Max Kleijberg, Andrea Kleeberg-Niepage

**Affiliations:** 1 Department of Learning, Informatics, Management and Ethics, 27106 Karolinska Institutet, Stockholm, Sweden; 2 The Center for Health Economics, Informatics and Health Care Research (CHIS), Stockholm Health Care Services (SLSO), Region Stockholm, Sweden; 3 Department of Psychology, 38982 European University of Flensburg, Flensburg, Germany; 4 Department of Global Public Health, 27106 Karolinska Institutet, Stockholm, Sweden; 5 Department of Neurobiology, Care Sciences and Society, 27106 Karolinska Institutet, Stockholm, Sweden

**Keywords:** adolescents, youth, young adults, community and public health, lived experience, scandinavia, qualitative methods: arts, art-based research, interdisciplinary

## Abstract

In this article, we explore the perspectives of 13–15-year-olds living in Sweden about the first wave of the Covid-19 pandemic, through inductive analysis of 187 of their drawings. Through reconstructive serial picture analysis, three types of meaning were derived: (1) *A new normal in dystopian scenery* points to the disruption of daily life and development of new praxis and meaning in a context of threat and restriction; (2) *Disrupted relationships* refers to these adolescents’ self-portrayal as solitary, without adult guidance or friends prominent; and (3) *Negative emotions and compliant behaviors* addresses a range of negative emotions and expressions of loss with few proactive strategies illustrated. General existential distress appears in these drawings, seemingly compounded by both developmental stage and other factors in addition to the pandemic context. Drawings suggest a restricted repertoire of ways of dealing with challenges confronting these adolescents, who seemed to feel left to their own resources.

## Introduction and Aim

Although children and adolescents were generally recognized to be neither those most vulnerable to nor the foremost drivers of Covid-19 infection in its’ first wave (Ludvigsson, 2020a; 2020c), it has been repeatedly acknowledged that they may be particularly affected by the pandemic and measures to contain disease transmission, especially in terms of psychosocial consequences (Chawla et al., 2021; Meherali et al., 2021). While studies relating to psychosocial effects of the pandemic on children and adolescents are prolific and continually increasing (see Chawla et al., 2021; Meherali et al., 2021), there are still notable knowledge gaps. Many studies are limited to needs of a specific group, often those with particular morbidities or life-situations (see Giannakopoulos et al., 2021; Mirlashari et al., 2021; Sharma et al., 2020; Tse et al., 2021). In addition, only a minority of studies are based on data generated directly by children or adolescents, with most instead relying on proxy reports from teachers, parents and other sources (Berasategi Sancho et al., 2021; Evans et al., 2020; Ferrari et al., 2021; Ferraro et al., 2021; Fitzpatrick et al., 2020). In general, the extant research is framed by professionalized knowledge and frameworks, with few open explorations of salient issues from the perspectives of children or adolescents themselves.

This notable absence of direct input from children and adolescents in the literature is in stark contrast to the development of New Childhood Studies, which understands children and young people as socially competent actors able to participate on equal terms in societal processes and dynamics, with perspectives valuable to contribute (Bodén, 2021; Jans, 2004; Kirk, 2007; Powell & Smith, 2009; Prout, 2011). Given the extent to which children and adolescents are affected by the Covid-19 pandemic, it becomes particularly relevant to recognize their knowledge by exploring and understanding their views of the world and their experiences. This is mirrored in several political, social, and research agendas, including the “United Nations Convention on the Rights of the Child” (1989, 1989).

One means of accessing children and adolescents’ perspectives is through analysis of their drawings, instead of relying on different forms of text-based data that more generally underlie qualitative research. There is a substantial body of literature arguing that drawings are a way for children of different ages to express their understanding and experience of their life worlds, in ways that reflect the surrounding culture and its values (Cox, 2005; Eriksson, 2014; Farokhi & Hashemi, 2011; Galvez et al., 2021; Gernhardt et al., 2013; Kleeberg-Niepage, 2016; Picard et al., 2007; Rübeling et al., 2011). An additional benefit is that drawing provides a form of expression regardless of degree of literacy or linguistic prowess. Picard et al. (2007) concluded that drawings were found to be more expressive and detailed as children age, although it can be argued that this is related to both interest in drawing as well as the significance of the subject matter for the child (Gernhardt, 2014; Richter, 1997). Analysis of drawings has been used to gain insight into children and adolescents’ views of themselves (Scheidecker et al., 2019), the future, (Kleeberg-Niepage, 2016; Kleeberg-Niepage & Marx, 2021) various health experiences (Eriksson, 2014b; Galvez et al., 2021), as well as their perspectives of the environment around them (Farokhi & Hashemi, 2011).

In this article, we aim to explore the perspectives of 13–15-year-olds living in Sweden about the first wave of the Covid-19 pandemic, through an exploratory, inductive serial analysis of their drawings. The drawings analyzed here were collected, archived, and made available to us by the Swedish Archive of Children’s Art (SBBA). SBBA is a public archive founded in 1977, which has collections of art created by preschoolers to 20-year-olds from as early as 1787 up to the present day (Martola, 2014). SBBA collects children’s visual artistic creations during significant societal events to be used in exhibitions and for research (Svenskt barnbildarkiv, 2021).

## Background

### The Swedish Context

In contrast to many countries, the Swedish pandemic strategy through June 2020 was based on voluntary, stepwise actions without a formal lockdown, relying on strong recommendations instead of legal, enforced restrictions (Kavaliunas et al., 2020). During Spring 2020, recommendations emphasized individual responsibility and included good hand hygiene; working from home when possible; maintaining physical distance to others; avoiding indoor social contacts; limiting non-essential travel; and staying home if any symptoms of Covid-19 were experienced (Bray, Blake, et al., 2021; Bray, Carter, et al., 2021; Ludvigsson, 2020b). Legislative regulation at this time related to international travel restrictions; restriction of visits to residential care facilities for elders; limits on the size of public gatherings; as well as some requirements for restaurants, schools, etc. (Ludvigsson, 2020b) Primary schools through grade 9 (ages 15–16) remained open with mandatory attendance, while secondary schools and universities held only digital lessons during Spring 2020; however, a small proportion of parents did keep their children home from school, primarily prior to mid-April 2020 (Ludvigsson, 2020b). Face masks were only recommended in health care and elder care and were not commonly used in Sweden at this time.

### Children and Adolescents’ Perspectives on the Covid-19 pandemic: Existing Literature

In general, systematic reviews (Chawla et al., 2021; Meherali et al., 2021) indicate that present knowledge about the impact of the Covid-19 and other pandemics on children and adolescents is primarily based on single group, cross-sectional studies, with data obtained indirectly rather than from the perspective of young people themselves. Meherali et al. (2021) point out that pandemics, including Covid-19, tend to lead to stress, worry, and a sense of helplessness, as well as social problems and risky behaviors. Chawla et al. (2021) conclude that the impact of Covid-19 on these age groups includes increased anxiety, particularly in girls and older adolescents, with a decrease in physical activity and quality of life along with increased sleep, screen, and internet time. Studies specifically focused on adolescents tend to emphasize this pessimistic perspective, particularly related to changes in schooling (Branquinho et al., 2021; Scott, S. et al., 2021; Scott, S. R. et al., 2021) and the loss of its’ relational function (Fioretti et al., 2020), as well as effects on friendships and family contact (Branquinho et al., 2021; Rogers et al., 2021; Scott, S. et al., 2021; Scott, S. R. et al., 2021), with a wide range of negative emotions cited in the literature. The impact of the pandemic on those already vulnerable is particularly noted (Lehmann et al., 2021; McCluskey et al., 2021; Scott, S. et al., 2021). However, Chen et al.’s longitudinal study from Sweden (Chen et al., 2021) comparing quantitative data from 584 15-year-olds from 54 schools in six municipalities, points to a need for caution when drawing conclusions about the effects of the pandemic. They compared baseline survey data generated prior to the pandemic with data from two follow-up survey timepoints, one before and one during the pandemic, finding surprisingly few differences between the follow-up groups. They conclude that the lower levels of happiness and increased stress and psychosomatic symptoms appeared related to increased age rather than the effects of the pandemic itself.

We found little empirical data specifically focusing on the perspectives of adolescents of the ages included in our study, as most studies include a broader age span (Branquinho et al., 2021; Fioretti et al., 2020; Lehmann et al., 2021; McCluskey et al., 2021; Rogers et al., 2021; Scott, S. et al., 2021; Scott, S. R. et al., 2021). Participants in studies are labeled as adolescents from as young as 12 years old (Lehmann et al., 2021) to as old as 24 (Branquinho et al., 2021). The extant research also derives from different settings, and thus from areas with different responses to the pandemic, including Brazil, Haiti, Norway, the UK, and the US. Only Chen et al.’s study—based on survey data—focuses solely on adolescents in Sweden (Chen et al., 2021). While most of the studies found are based on questionnaire or verbal data, a few include drawings, although these derive from children aged 6–13, that is, generally younger than those in our sample (Abdulah et al., 2020; Amrutha et al., 2021; Bray, Blake, et al., 2021), with one study exclusively from Iraqi Kurdistan (Abdulah et al., 2020) and another from India (Amrutha et al., 2021). These drawing-based studies, mostly in pre-adolescent children, found relatively positive pictures (Abdulah et al., 2020; Amrutha et al., 2021), depicting home as a place of safety during the pandemic, with mothers and siblings present (Abdulah et al., 2020). A Spanish study instead used photovoice as part of an online data collection among 12-year-olds, focusing specifically on photos of both the most comfortable spaces and tele-study spaces at home, thereby gaining insight into the value of space for family encounters (Cuerdo-Vilches & Navas-Martín, 2021). An additional international drawing-based study included 7–12 year old children from Australia, Brazil, Canada, Spain, the UK, and Sweden (Bray, Blake, et al., 2021). This study requested drawings specifically related to one question, about “why we are all trying to stay at home during the coronavirus” (Bray, Blake, et al., 2021). The researchers concluded that most participants had good knowledge about Covid-19 transmission, measures to decrease virus spread, places of safety, and the role children themselves might play in virus transmission (Bray, Blake, et al., 2021). The same group’s survey-based publication on Covid-19 health literacy (Bray, Carter, et al., 2021) found that while parents were the predominant source of health information according to children in the other included countries, this was not the case in Sweden, where children reported school as the main source of information, followed by parents. However, the Swedish parents themselves believed they were the primary source of Covid-19 information for their children. It is also notable that when children in Sweden were asked about their preferences for information, both school (36%) and TV/TV news (24%) were ranked higher than parents (14%). This is in striking contrast to children in the other countries, with school only otherwise listed by children in the UK as a preferred option for information, although to a lesser extent (9%).

## Methods

### Data Collection

Museums and archives play a unique societal role through their mandate to collect, preserve, mediate, exhibit, and research material and immaterial culture and the human world of the past, present, and future (Regeringskansli Swedish Government, 2017). In line with recommendations from the International Council of Museums to support community resilience through rapid response collecting and documenting of the pandemic and its impact (International Council of Museums, 2020), SBBA initiated a call for the collection of children’s visual art related to the Covid-19 pandemic in March 2020. The call was disseminated through their website, social media and mailing lists, as well as their existing network of schools and teachers, children’s culture centers, and Swedish county art consultants. This collection was in accordance with European Union General Data Protection Regulation (Vollmer, n.d.), with archived data allowed to be used for academic research. SBBA is responsible for the handling of all personal data.

Those contacted were asked to spread the call through their wider networks. The call invited anyone up to 20 years old to create and submit any kind of visual art about Covid-19 to SBBA. The contributors were asked to depict their experiences based on what the pandemic situation feels like, looks like, and what is different now, as well as prompted to share anything else that they would like to include. SBBA asked the children to provide background information including their name, gender, age, location in Sweden, and when and where they created their submission (e.g., at school, or at home). Between April 15 and July 31, 2020, SBBA received 1154 submissions for this Covid-19-themed collection, created by children aged 4–16. Nearly all were made in school and submitted by teachers. No further information is available about the process in the classroom setting.

### Data Analysis

The data analysis process began with several visits to the archive to examine the full collection in late 2020. All submissions were repeatedly reviewed, and it was noted that most were drawings. We initially chose to focus on adolescents aged 13–16 as we expected a richer variety of experiences, phenomena, and impressions to be depicted by this group than in drawings from younger children, as supported by researchers and theorists in art education (Lowenfeld & Brittain, 1982) and developmental psychology (Picard et al., 2007). This was also in line with our impressions from the initial perusal of all the drawings in the collection. Initial sample selection was thus based on all 211 submissions from this age group. As only four pieces were by 16-year-olds, we limited the sample to 13–15-year-olds, an age bracket corresponding to the last years of obligatory schooling in Sweden. Only one submission was not a drawing but a collage and was excluded, as were those that lacked background information. This led to a final sample for analysis of 187 drawings made between early April and mid-June 2020.

The drawings were analyzed using an inductive three-step methodological approach for serial picture analysis based on a qualitative, reconstructive research paradigm (Bohnsack, 2009, 2010). Initial analysis was begun by the team working with Covid-19 data in Sweden, with backgrounds in public health, nursing, palliative and end-of-life care, and design. The original team recognized a need for additional methodological and substantive expertise, and therefore contacted two researchers experienced in reconstructive serial analysis of children’s pictures, with backgrounds in developmental and social psychology. This five-person interdisciplinary group had not all worked together prior to collaborating in this project. In this project, we worked solely through virtual meetings.

The reconstructive approach applied here derives from art history (Imdahl, 1996, (orig. 1979); Panofsky, 1972) and rests on the assumption that every human activity, including drawing, corresponds to and represents different layers of knowledge and meaning (Mannheim, 1964), that is, meanings that are explicit and readily communicable as well as those that are latent and implicit. Reconstructive approaches are particularly well-suited for addressing latent meaning based on subjects' (embodied) experiences, implicitly learned through everyday life and practices, in a specific socio-cultural context. Such experiences are rarely conscious and therefore not communicated explicitly. This means that as reconstructive methods aim to focus on implicit meaning, the analysis process progresses from analyzing *what* is communicated (motif) to reconstructing *how* it is communicated (the contextualized meaning) in a series of several steps, explained below.

**Figure 1.**
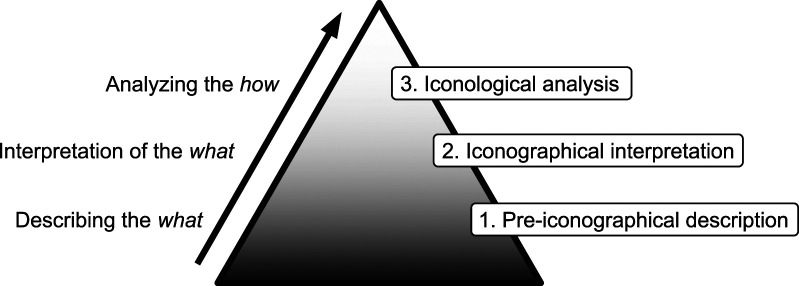
Three-step approach to analysis based on Panofsky as applied here.

The analysis conducted here follows Degen & Kleeberg-Niepage’s (Degen & Kleeberg-Niepage, 2021) reconstructive serial picture analysis, inspired by Pilarczyk and Mietzner (2008). This approach applies the principles of Panofsky, 1972 iconological three-step approach to analysis of a series of pictures, rather than single images, and is shown in Figure 1.

In the first “pre-iconographical” step, each drawing is described exclusively in terms of the shapes, sizes, positions, colors, and materials that comprise the drawing. We used this descriptive step, carried out by one researcher in the original team, to formulate a comprehensive portrayal of each individual picture in the full sample in terms of “what” the content of the picture consisted of, avoiding interpretation in this stage.

**Figure 2.**
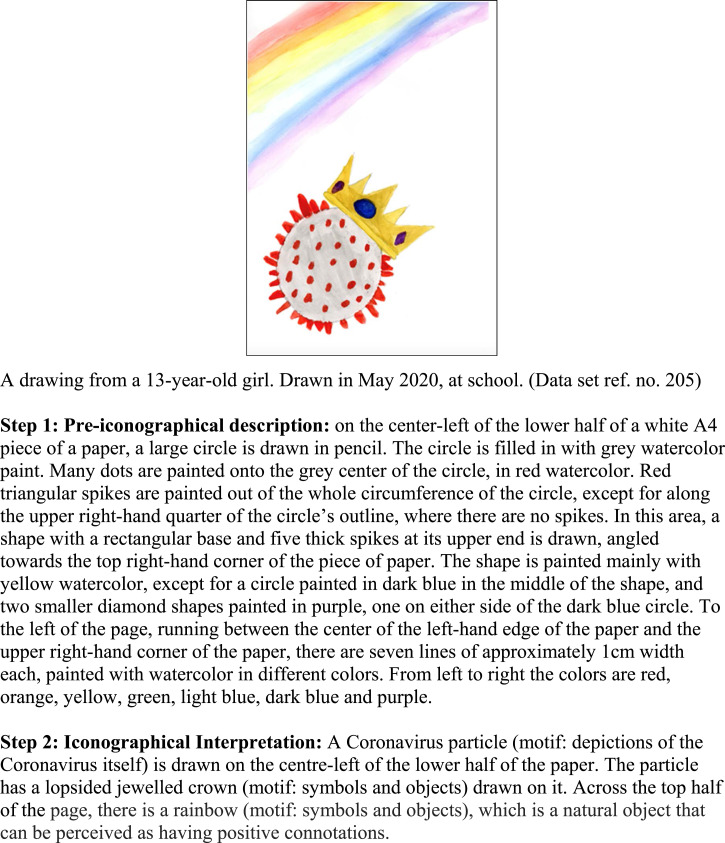
Example of the first two analytic steps for one picture.

In the second step, “iconographical interpretation” (Panofsky, 1972), we added contextual interpretation of the content represented by the characteristics described in the pre-iconographical step. This step was carried out by the same researcher who conducted the first step and reviewed and discussed in the full team. Figure 2 provides an example of the first two analytic steps, illustrating this progression from description to the start of a more interpretative process. We continued by comparing drawings in the series for similarities and differences, resulting in an iconographical typology consisting of six types of motif-based descriptions: (1) symbols and objects; (2) depictions of the coronavirus itself; (3) people; (4) places; (5) depictions of the planet Earth; and (6) references to time, or specific timepoints (listed by decreasing frequency in the series). Each picture could contain one or more motifs. These motifs were discussed and agreed upon in the full research team and are shown in Supplemental Appendix 1.

The third step, “iconological analysis,” marks the shift from *“what”* in terms of motifs to *“how,”* with a focus on implicit meaning in the full series of drawings. This iconological typology goes beyond and cuts across the explicit motif-based descriptions in the second step, to reconstruct an implicit layer of meaning (Mannheim, 1964). This interpretive analytic step initially was conducted independently in two different constellations of the involved researchers. We then constructed a matrix to confront similarities and differences in each initial typology. We thereafter integrated and elaborated these separate conceptualizations into a common formulation, making certain that all analytic points were included. This process took place through discussion in the research team, resulting in one cohesive iconological typology, which was continually elaborated during the joint writing process. The full team finalized the typology presented here together.

To facilitate the understanding of analytical points presented in our findings, we share some of the adolescents’ drawings in this results section and in the supplemental appendix. We have made efforts to choose pictures that could illuminate several points and illustrate variation in styles and content indicating the breadth of the data base; pictures may be relevant in one or more of the types of meaning presented below. Since the images are in the possession of a public authority, and so in the public domain, they can be used and shared without infringing European intellectual property laws (European Union Intellectual Property Office Observatory, 2021). To protect the adolescents’ privacy, we minimize the amount of personal information shared when presenting pictures.

## Findings: An Iconological Typology

The process of analysis resulted in a typology consisting of three types of overarching meanings interpreting the implicit knowledge and incorporated experiences of the adolescents. As noted above, these interpretative iconological types of meaning cut across the iconographical typology’s six motif-based descriptions (See Supplemental Appendix 1). While the types of meanings in the iconological typology are not mutually exclusive, and one or more types of meaning can be relevant for each individual drawing, they are presented separately here. References to empirical data examples are provided for analytic clarity; these are referred to either by drawing number in Supplemental Appendix 1 or included as an illustrative example in the narrative text.

The first type of meaning, “A new normal in dystopian scenery” is based on images that address threats to life and changes in everyday life on both micro and macro levels, depicting the development of a new normal in response to the pandemic. The second type of meaning, entitled “Disrupted relationships,” refers to the adolescent as a social actor, in relationship to themselves, those close to them, and the surrounding social context. The last, “Negative emotions and compliant behaviors” is response-related, encompassing images portraying person-related—both emotional and action-oriented—responses to the pandemic. We present these themes in detail below.

### A New Normal in Dystopian Scenery

**Figure 3.**
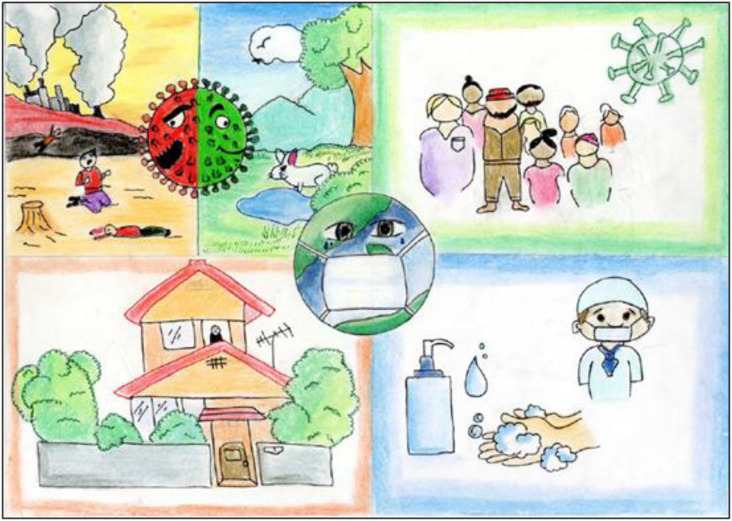
(Data set ref. no. 857). 13-year-old girl, May 2020.

**Figure 4.**
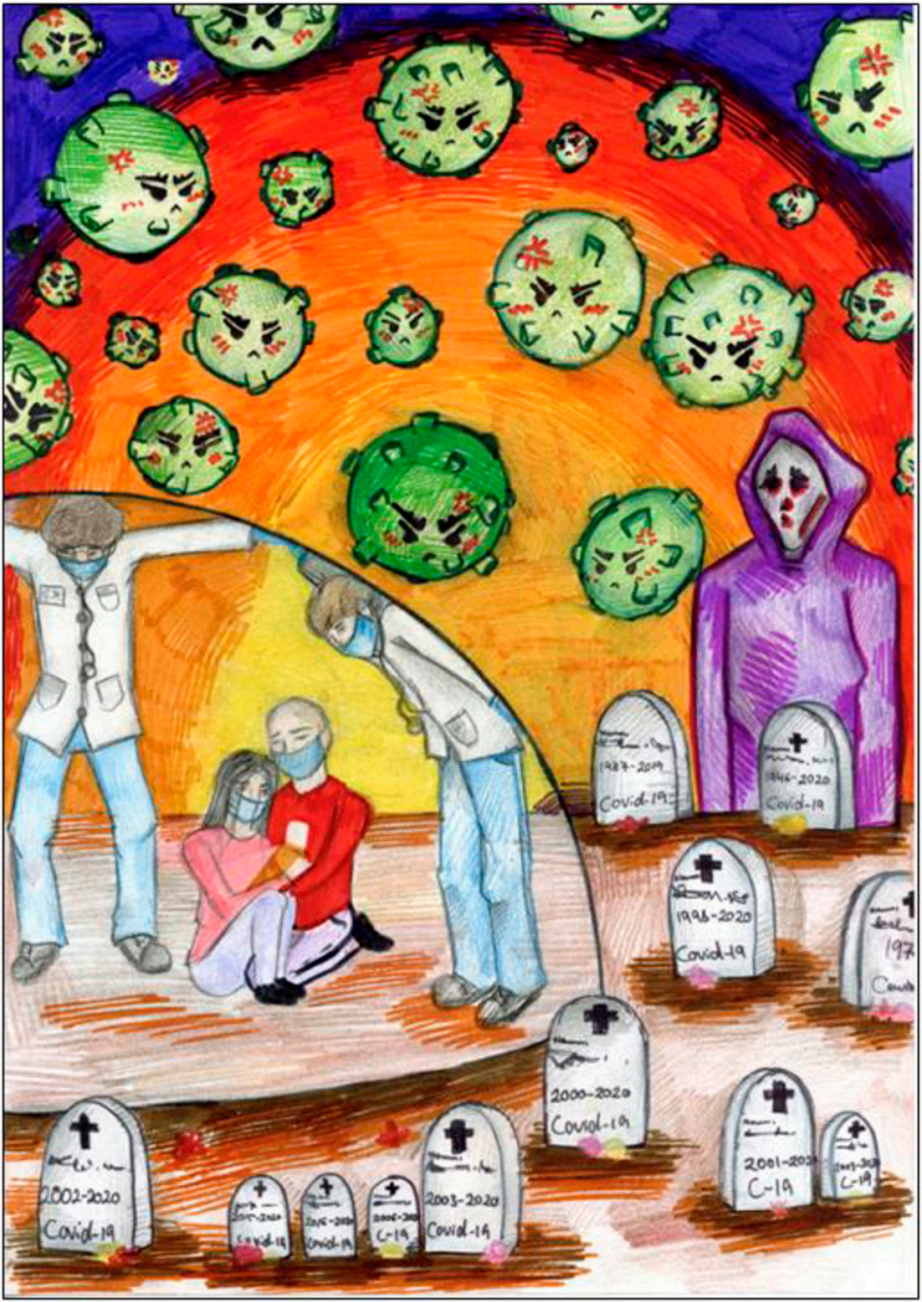
(no. 869). 14-year-old girl, May 2020.

**Figure 5.**
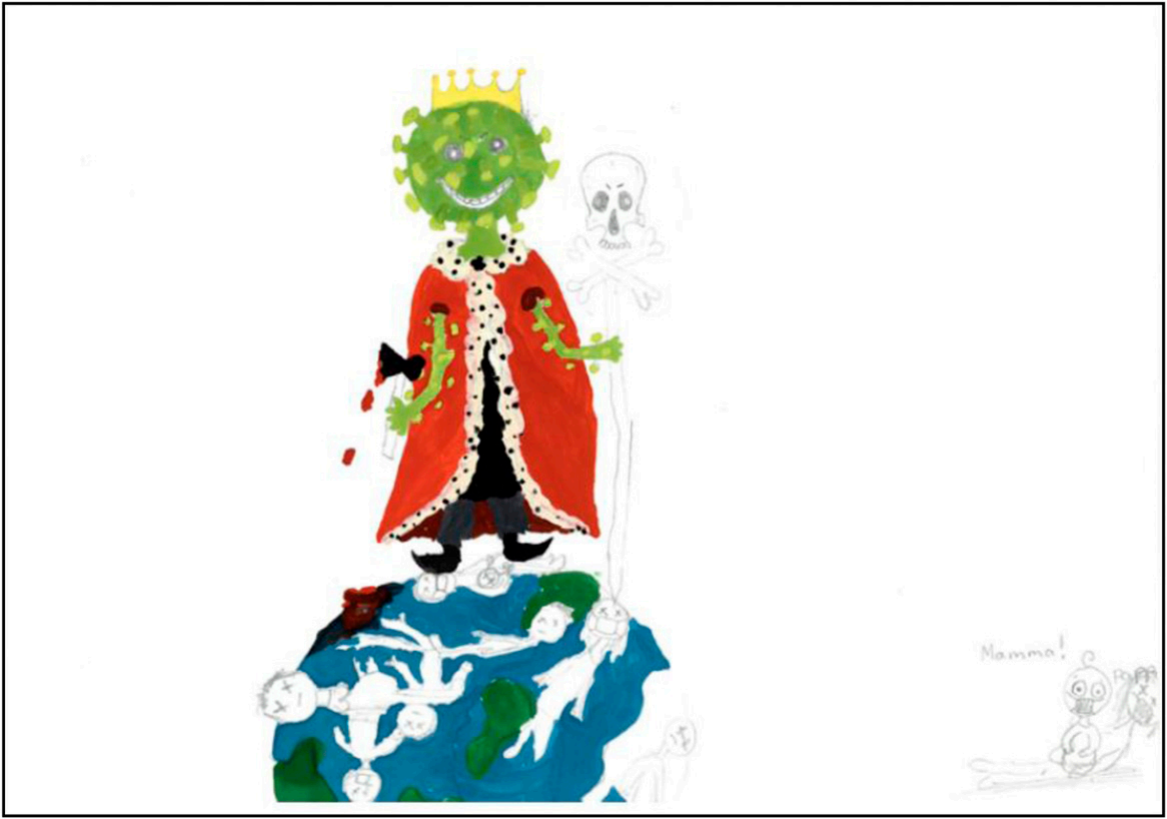
(no. 680). 15-year-old girl, May 2020.

In this type of meaning, fundamental changes are addressed, both at the macro level—referring to, for example, humankind and planet Earth, and at the micro level—referring to individual daily life and interactions. Threats to life, both one’s own and life on Earth in general, are often expressed in dystopian (See Figure 3) and apocalyptic scenarios (#41). While some pictures depict threat as general (#41), others are more specific, with fellow human beings shown as threatened on either an individual (#11) or collective level (#27). People of all ages are subject to threats to their lives, as seen in the ages on gravestones (Figure 4) and in the small image of what appears to be a lone baby with a dead parent, in the periphery of Figure 5. Social behaviors previously accepted or unnoticed are reevaluated and illustrated as dangerous or forbidden, for example, hugging, kissing, and close contact with others (#11). Humanity as a whole also appears threatened, not only by the Covid-19 pandemic (#10), but also by other environmental (see Figure 3, upper left) and political (#23) phenomena. The virus is often shown wearing a crown and characterized as a deliberate and personified actor that threatens and refuses to acknowledge humans’ assumptions of their superior role (Figure 5). Instead, both planet Earth and humanity are portrayed as shrunken and victimized in relation to the Covid-particles (#43), which are given human characteristics with a range of emotions and appear determined in their attack on the Earth and its inhabitants. Thus, an altered hierarchy of power in the world appears to be portrayed.

**Figure 6.**
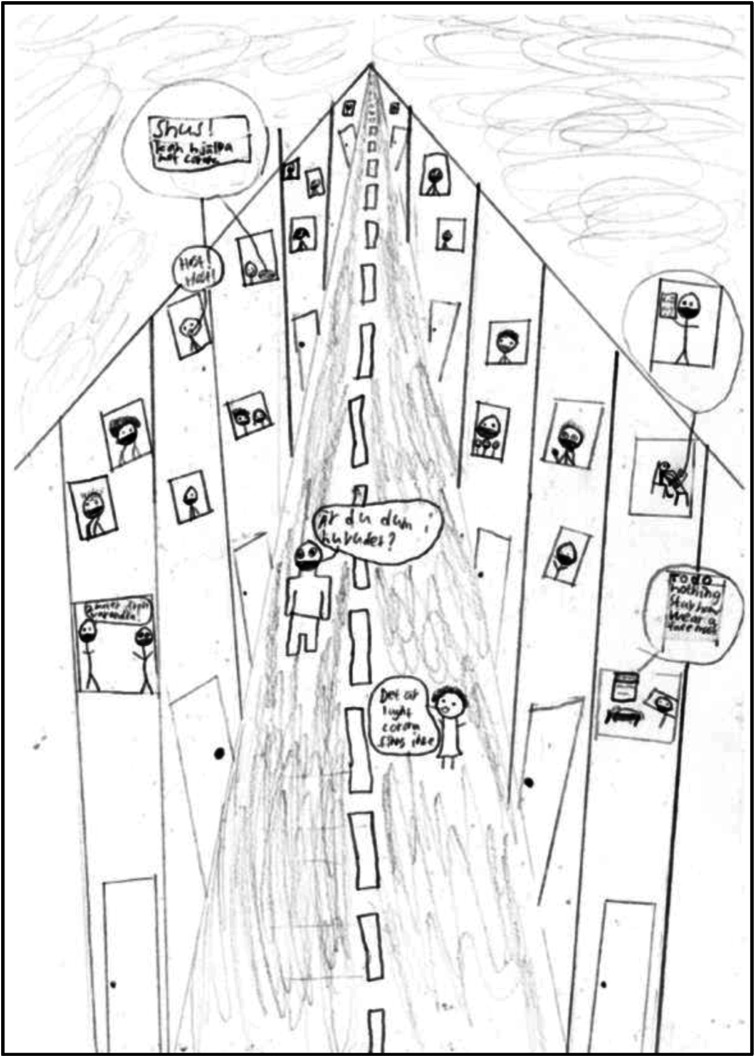
(no. 684). 14-year-old boy, June 2020.

These adolescents’ drawings include numerous implicit and explicit moral judgments. These are in part about environmental and political issues, but are predominately seen in dichotomous distinctions between behaviors portrayed as endangering (e.g., being outside) versus protective (e.g., staying at home). Figure 6 offers a clear example, as the person in front in the street is drawn as saying “It’s OK, corona doesn’t exist,” while the person behind them comments “Are you stupid?”

During the pandemic, new normal conditions for everyday life are created in which objects and behaviors have altered meanings. As noted above, this occurs through common behaviors becoming morally reevaluated and symbolically loaded. Relationships to objects are also changed, as illustrated in pictures of the hoarding of toilet paper and food products (as in the empty shelves in #54). Objects and people specific to health care but not part of daily life—and without much attention or moral significance before the pandemic—that is, disinfectant hand-gel, face masks (#4) and health care professionals (#28)—now illustrate not only the new normal, but also become symbols for behaviors perceived as morally righteous. New daily life is also portrayed in relation to loss, for example, of previously taken-for-granted comforts, activities, and travel (e.g., #40), and public spaces are portrayed as empty (#32). As succinctly illustrated in Drawing #32 (Supplemental Appendix 1) showing an empty bench with an admonition to keep distance with the text “can’t corona disappear please…miss the regular old days,” the new normal is generally not depicted positively.

### Disrupted Relationships

Pictures here address a wide range of relationships affected by disruption, loss, restriction, threat, insecurity, and isolation. The relationships depicted regard individuals, sometimes explicitly pointed out as the adolescent themselves, their proximal social circles, as well as a broader societal context.

**Figure 7.**
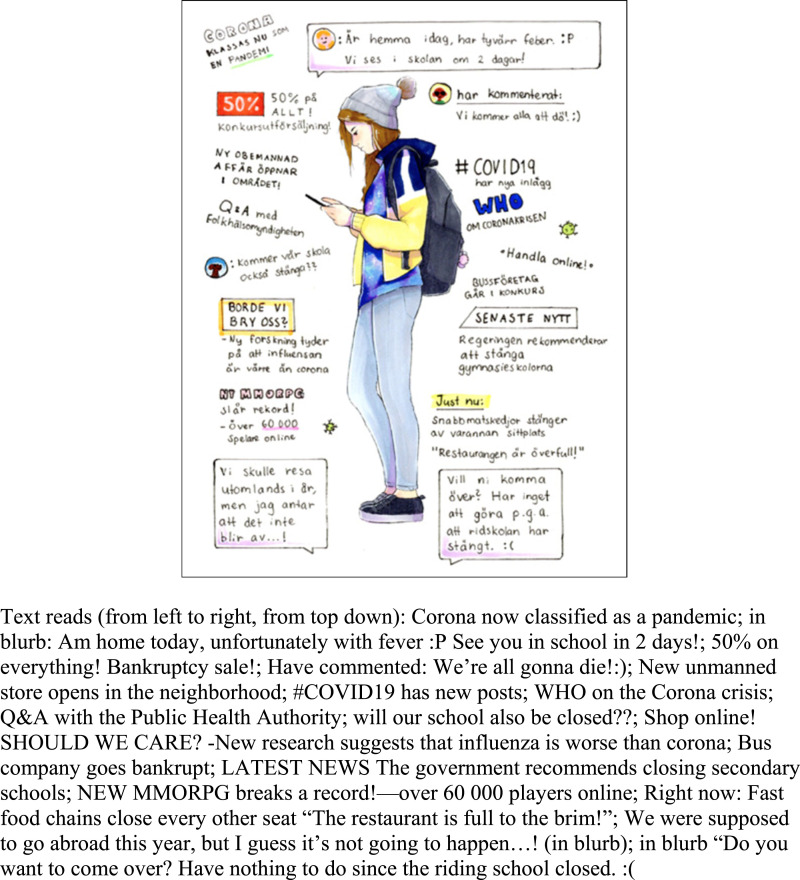
(no. 290). 14-year-old girl, May 2020.

The drawings tend to demonstrate singularization, with one person alone in a situation, as exemplified by #48. This drawing also points to a common feature of this apparent loneliness in the pictures, with few illustrations of fostering connections through virtual channels. With the exception of the drawing shown in Figure 7, we found little depiction of what we would have defined as virtual social connectivity, although a number of devices are shown as important for life in the pandemic (#4), generally as a means of entertainment. While gaming or the use of digital devices and apps might indeed include others, the potential others are not depicted in these pictures. As researchers we were struck by the lack of relationships to other people focusing on closeness rather than distance in most drawings. We noted little portrayal of what we recognized as non-professional care for others, with few elders or family members drawn. Endangering conditions are generally shown as threats to one individual or in a more global perspective, with a notable absence of friends and family in these drawings (see Figure 5 and #53 for the exceptions).

Intersubjective relationships in a broader social context, that is, in groups and nations, show both new pandemic-related as well as long-established forms of representations and stereotypes. Covid-19-related immobility and geographical confinement is shown in a variety of ways (e.g., #39), with differences between countries depicted. Such images often refer to public discourse about how the Covid-19 situation is negotiated, for example, the pandemic’s early impact on Italy (#37, #38), Trump’s role in the world (#23), or Sweden’s particular strategy in fighting the pandemic (#37). China (e.g., as symbolized by its flag) is thematized in several pictures as the place of origin or cause of the pandemic (#35, #36). Most of these images also appear to reflect then-ongoing societal discourse about the cause of the disease, sometimes referring to specific living and eating habits, as in #36, which includes text reading “don’t eat bats! (and don’t eat anything else strange for that matter)”. A small minority of drawings suggest a political stance which did not accept this view. Drawings #21 and #22, for example, both show a person appearing to have Asian characteristics with the text “I am not a virus”; these are among the few drawings illustrating a critical perspective about the consequences of blame for individuals.

### Negative Emotions and Compliant Behaviors

**Figure 8.**
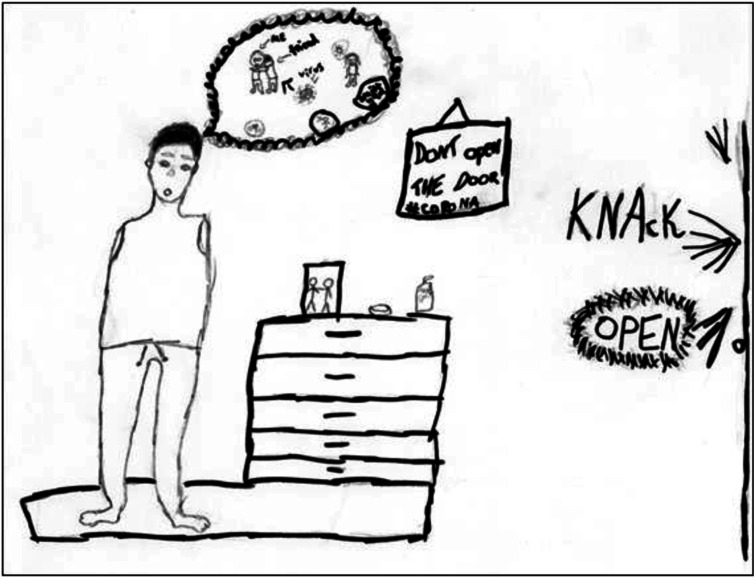
(no. 913). 14-year-old boy, May 2020.

Pictures here depict these adolescents’ ways of being during this first pandemic phase, including both emotional and behavioral responses. The feelings represented appear predominately negative, with subjects seeming sad, aggressive (#34), worried, anxious, and tired of or from the situation. For example, nurses appear worn (#28), and picture #53’s text ‘missing you grandma’ indicates sadness and a sense of loss. The picture in Figure 8 exemplifies a gap between desire and reality perceived in the pandemic context. The picture shows what appears to be a boy alone with amputated arms, imagining hugging a friend, but with Covid-19 portrayed as a threat appearing to make him incapable of doing so. In the same drawing, a small picture on the bureau shows people together, while one hanging on the wall warns not to let people in because of Covid, at the same time as there is a knock on the door.

The resulting mode of being is characterized by examples of dealing with the pandemic situation in a compliant, rather than rebellious manner. These pictures suggest a situation in which life is “on hold,” with these adolescents drawing scenes of everyday life focusing on online-entertainment with loss of many other (physical) activities, for example, #17 is one of several drawings showing sports as restricted. Picture #20 is a notable exception, illustrating a boy alone wearing a facemask and playing basketball, with a virus particle as the ball. Human happiness seems to be a rare exception in these pictures; when illustrated it is most often shown in relation to acting in accordance with rules about prescribed distance (see #6 and #9). This can be contrasted to the depictions of positive emotion and deliberate action drawn in relation to the Covid-particles, as noted above.

## Discussion

This article is based on an inductive, systematic stepwise serial analysis of 187 drawings created during the 1st wave of the Covid-19 pandemic by 13–15-year-olds in Sweden. These adolescents were asked, generally in a school setting, to depict their experience of the pandemic. During analysis and interpretation of findings, we integrated our disciplinary perspectives, differences, and distance into shared vocabularies and ideas to formulate three overarching types of meaning that represent the adolescents’ implicit knowledge and embodied experiences of the pandemic, as depicted in these drawings. The first type of meaning, *A new normal in dystopian scenery*, points to both the disruption of daily life on macro-and micro levels leading to the development of new praxis and meaning in a context of threat and restriction. The second type, *Disrupted relationships,* found singularization of these adolescents, nearly always portrayed without significant others. There appears to be a general tension in portrayals of life as quite solitary during the pandemic, while adolescents also depicted themselves as global beings, aware of, connected, and reacting to the world beyond their immediate social circles. The third type of meaning, *Negative emotions and compliant behaviors*, found few positive expressions of feelings other than when complying with rules, coupled to an array of expressions of loss with a limited range of coping strategies illustrated. In general, we found that the pictures to illustrate a notably restricted repertoire of ways of dealing with challenges that appear to confront these adolescents. Drawings suggest agency illustrated in terms of compliance rather than the resistance one might associate with adolescence, with focus on what they are not able to do, rather than on what they are doing. This is the case in these data, not only in dealing with the many changes in daily life and new practices brought about by the pandemic, but also in dealing with other challenges related to adolescence in a changing world, at present and regarding anticipated threats for the future.

There are some features of these data to consider when interpreting the findings. The adolescents who drew the pictures come from a wide range of geographical locations across Sweden, with varying socio-demographics. However, it should be recognized that the drawings were all created during the first pandemic wave, and this study is limited in that we lack further information about the situation of these adolescents before or after. Nearly all drawings in our study were created while at school, although we have no in-depth insight into this process beyond the questions provided to trigger the drawings. This context should be considered in relation to both norms in a classroom setting, as well as potential pressure to be in line with one’s peers, without making oneself too vulnerable in terms of self-revelation in the drawings.

In their Swedish study of adolescents before and during the pandemic, Chen et al. (2021) found few Covid-19-related mental health differences, concluding that this may be due to Swedish schools remaining open. However, in our data, we found notably few drawings illustrating the “relatively normal life” Chen et al. (2021) argue was still possible in the Swedish context, without formal lockdown implemented. Chen et al. (2021) also found that 13–15-year-old adolescents generally had lower levels of happiness and higher levels of both stress and psychosomatic symptoms than the other age groups in their survey, arguing that their stage of development, rather than the pandemic alone, may play an important role. In this phase of life, social bonds shift through differentiation of oneself from parents and family with increased validation of self through peers (Harter et al., 1997; King, 2013; McGue et al., 2005). In some ways, the Covid-19 pandemic appears to play havoc with such developmental processes, as these adolescents drew themselves in what often seems to be a social void, without parents, siblings, relatives, or friends clearly placed in drawings. We have seen no similar data reflecting this absence of adult support persons in the Covid-19 literature on children and adolescents. It may well represent an appropriate developmental trajectory in this age group, as studies of younger children in the pandemic tend to comment the positive presence of family, often illustrating home as a place of safety during the pandemic (Abdulah et al., 2020; Amrutha et al., 2021; Cuerdo-Vilches & Navas-Martín, 2021). The only other study we found that included drawings by Swedish participants makes no mention of how the somewhat younger (7–12-year-old) Swedish children in their international study portrayed home and the family (Bray, Blake, et al., 2021). However, Bray, Blake, et al. (2021) point out that the children from Sweden drew pictures associating the outdoors with safety, whereas children in the other participating countries instead drew their homes as places of safety. Our data, however, are more mixed in terms of illustrating a “stay at home” message, without directly illustrating home as a refuge. This may in part reflect differences in recommendations across countries, with less population-dense Sweden encouraging being outside with distance to others, rather than a strict homebound lockdown (Ludvigsson, 2020b). These studies, along with our data, suggest an interaction between local pandemic context and developmental stage.

Not only are adults strikingly absent, but there are also few peer figures in the pictures. This is quite notable, given the usual orientation towards peers in this developmental stage (Brown & Larson, 2009), and given that schools for this age group remained open in Sweden. Despite this, the overall impression in this serial analysis is one of solitary adolescents, left to their own resources. One may speculate if the pandemic accelerated a process of individualization, or if this is a Covid-19-related phenomenon.

We also interpret these drawings as suggesting a restricted sense of agency on the part of these adolescents, with social responsibility primarily shown in terms of following guidelines. We note few expressions of either differentiation, as noted above, or of resistance. The disrupted world of Covid-19 may well limit the growing autonomy usually associated with adolescence. Even in the few pictures which suggest that an active political stance challenging implicit norms has been taken, there is little sign of actual resistance in terms of testing boundaries or breaking rules.

This is not to imply that these adolescents are not aware of the world around them, quite the contrary. It is also evident that media and other societal discourse shape the experiences depicted in these drawings. For example, the image of the virus particle in many drawings is one familiar from global media. Additionally, depictions of facemasks are frequent and store shelves are drawn as empty, although masks were not recommended in Sweden until long after these pictures were drawn and Sweden did not suffer from the severe lack of products (e.g., toilet paper) seen in other countries. Picture texts could be in English, as well as in Swedish. There is obvious awareness of different pandemic conditions in different countries as well. We also note the appearance of long-standing hierarchies related to blame, for example, othering of the Chinese as a foreign threat. Other group distinctions seem newly constituted, including polarizations between older (as threatened) and younger people, or between those following regulations and those defying them.

Numerous pictures also appear to implicitly or explicitly link the pandemic to human-made ecocide, with scenes depicting a general dystopian state of the earth and life upon it. Human beings are shown as both being capable of causing disease and destruction, while on the other hand, seem helpless in the wake of these (multiple) crises. The state of the earth itself appears cause for concern in many pictures, as it is confronted with numerous threats, although other scenes of nature are among the few subjects in these drawings that suggest a sense of comfort or harmony. “Solastalgia,” that is eco-anxiety or distress caused by environmental change (Albrecht et al., 2007) becomes relevant here, as several scholars point to the importance of experiencing powerlessness and a lack of control over such changes (Albrecht et al., 2007; Degen et al., 2021). Marazziti et al. (2021) recently argued the need for more attention to be given to how the interaction between climate change, pollution, and the Covid-19 pandemic affects mental health, as all point to weaknesses in our ecosystem and our inability to protect ourselves. It should be remembered that Greta Thunberg, a Swedish adolescent environmental activist, played a prominent role just prior to the outbreak of the Covid-19 pandemic. This makes the absence of activism in these drawings even more noteworthy, but at the same time points to the importance of role models to help envision alternative possibilities and practices (Pihkala, 2020).

In conclusion, there appears to be general existential distress illustrated in the drawings in this data set. We argue that this may be related to a variety of factors in combination, with Covid-19 functioning to accelerate some pre-existing tendencies. Humans tend to seek solace in connection, while connection to others in the Covid-19 context is fraught with danger. Nature, often a source of comfort in Sweden (Stålhammar, 2020), also appears to be endangered by human behavior. A sense of powerlessness appears to result, with few options illustrated by these adolescents during the first pandemic wave. It is possible that adaptation and application of a broader range of options did develop later in the pandemic. However, these data highlight a need to further support this potentially vulnerable age group in maintaining positive connections and active means of dealing with the challenges confronting them and us all.

## Supplemental Material

Supplemental Material - A Qualitative Serial Analysis of Drawings by Thirteen-to Fifteen-Year-Old Adolescents in Sweden About the First Wave of the Covid-19 Pandemic Click here for additional data file. Supplemental Material for A Qualitative Serial Analysis of Drawings by Thirteen-to Fifteen-Year-Old Adolescents in Sweden About the First Wave of the Covid-19 Pandemic by Carol Tishelman, Johanna L. Degen, Sofía Weiss Goitiandía, Max Kleijberg, and Andrea Kleeberg-Niepage in Qualitative Health Research
