# Supplementary material for: A Qualitative Serial Analysis of Drawings by Thirteen-to Fifteen-Year-Old Adolescents in Sweden About the First Wave of the Covid-19 Pandemic
Source: Qual Health Res. 2022 May 21;32(8-9):1370–85. doi: 10.1177/10497323221101978 (PMC9350847; doi:10.1177/10497323221101978)
Supplement: Supplemental Material - A Qualitative Serial Analysis of Drawings by Thirteen-to Fifteen-Year-Old Adolescents in Sweden About the First Wave of the Covid-19 Pandemic [file sj-pdf-1-qhr-10.1177_10497323221101978.pdf]

Appendix 1 – Results of the iconographical analysis

Examples of drawings from each of the six motifs are presented here.

Motif 1: Symbols and objects

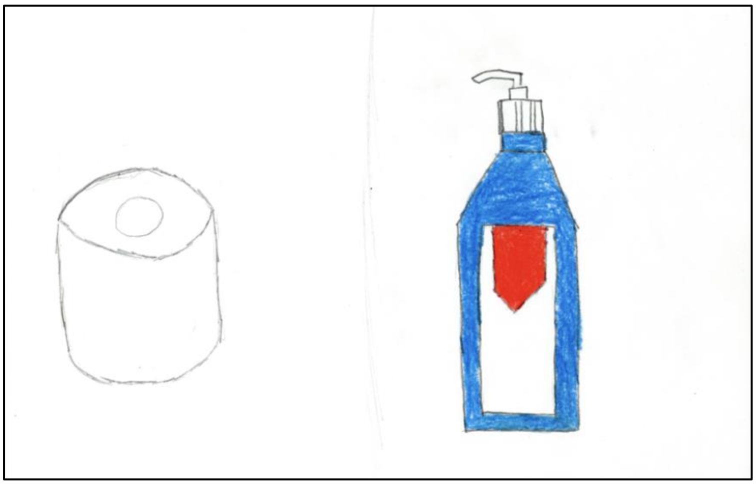

Drawing 1 (Data set ref. no. 761). 13-year-old boy, June 2020.

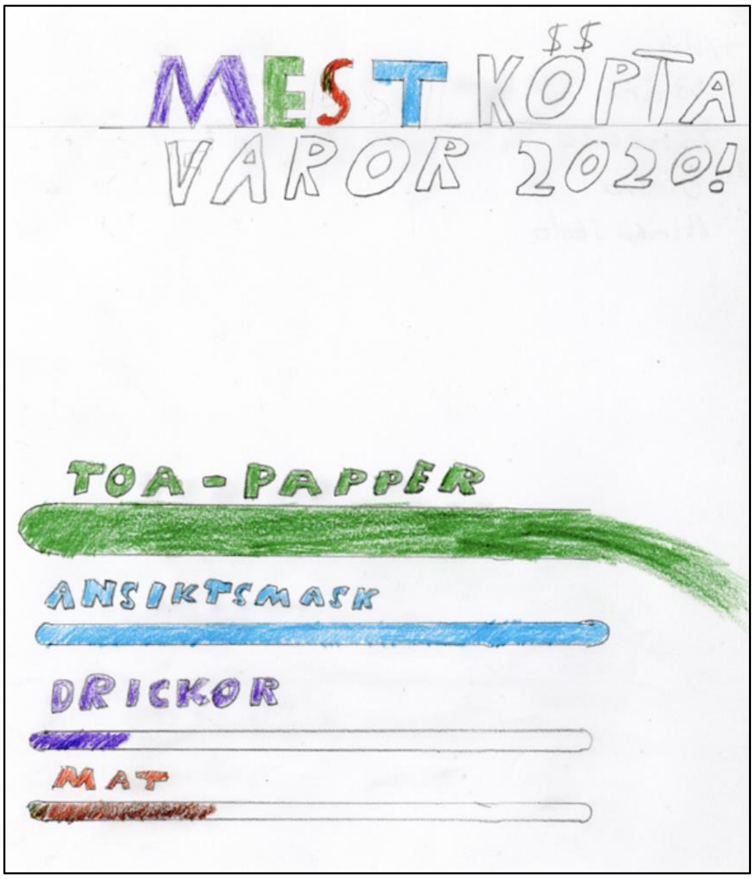

Drawing 2 (no. 783). 13-year-old boy, June 2020.

The title reads “most bought items 2020!” and the bars depict, from top to bottom, sales of “toilet paper” “facemasks”, “drinks”, and “food”.

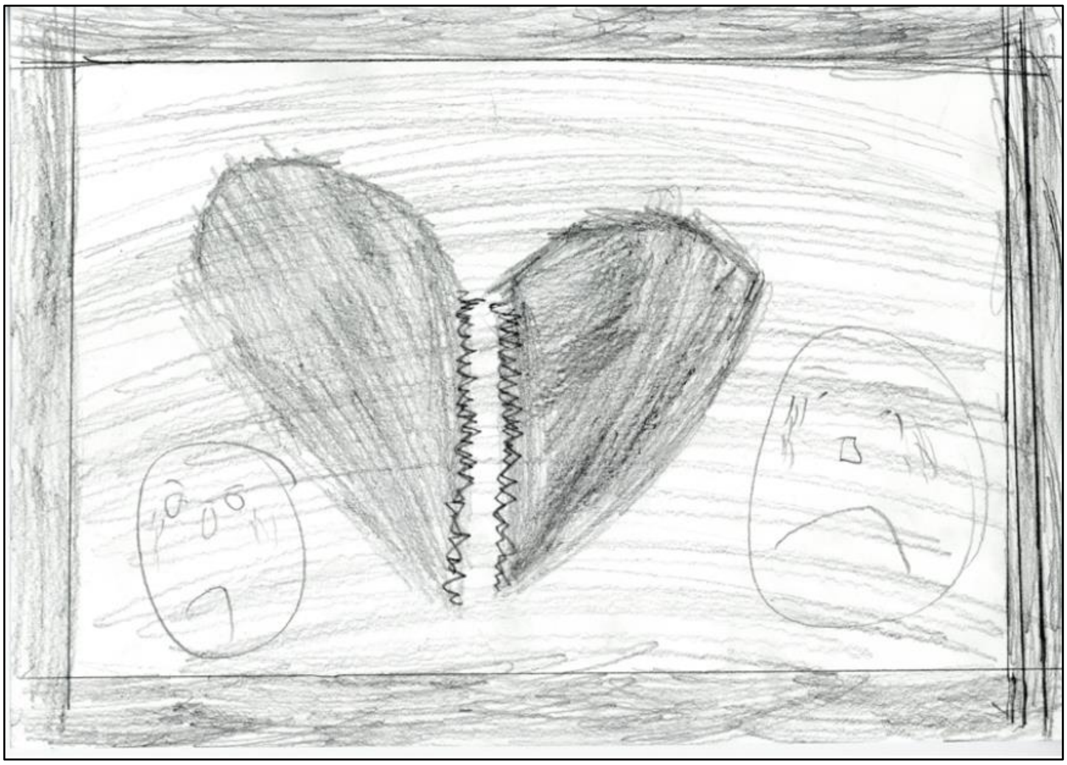

Drawing 3 (no. 791). 14-year-old boy, June 2020.

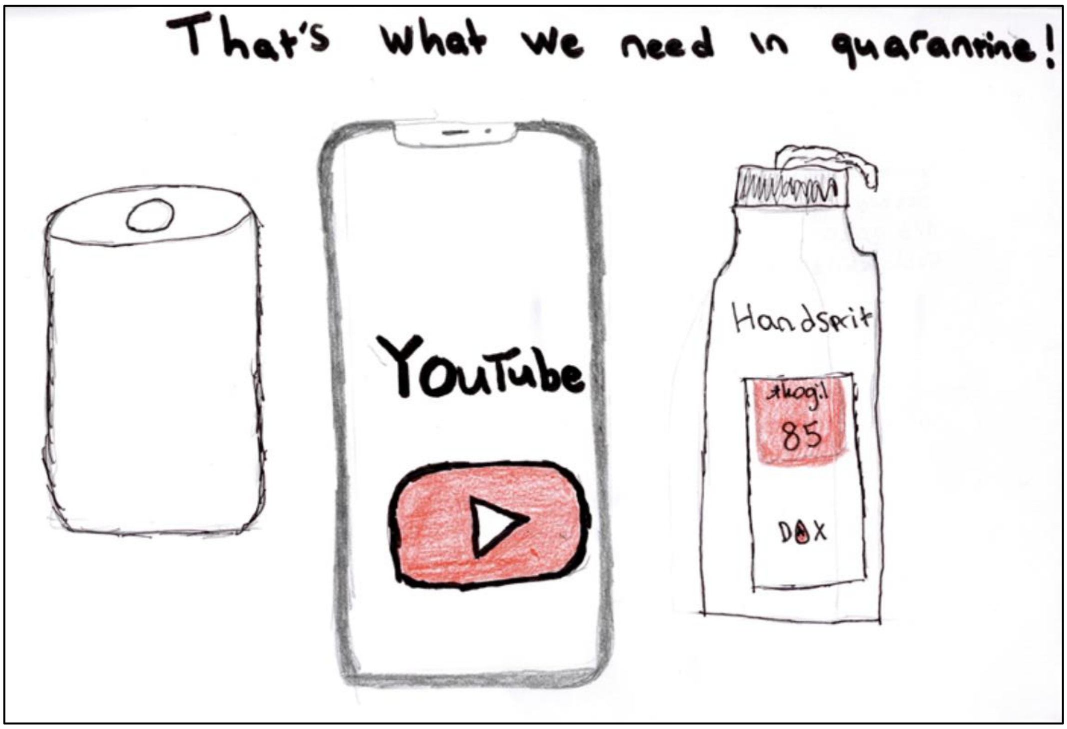

Drawing 4 (no. 707). 14-year-old girl, June 2020.

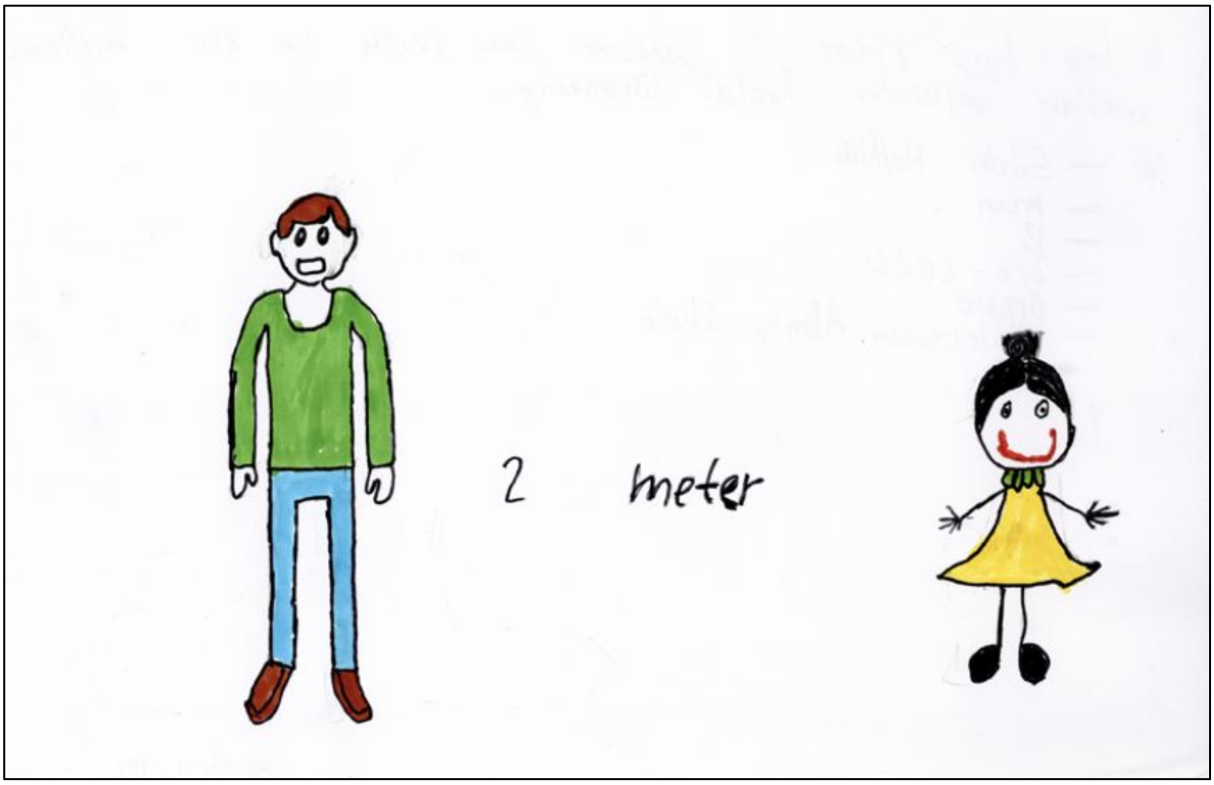

Drawing 6 (no. 784). 13-year-old boy, June 2020.

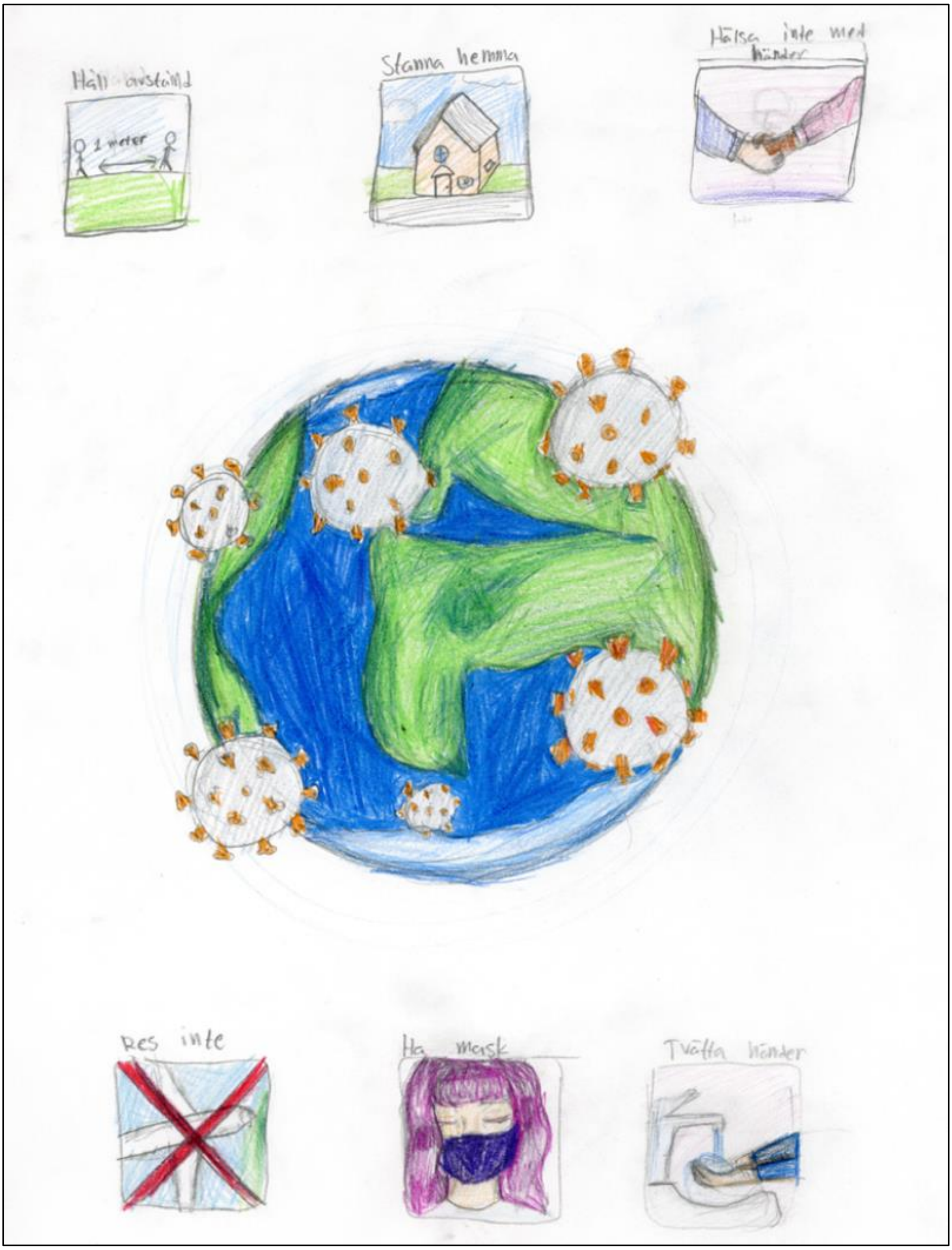

Drawing 7 (no. 917). 14-year-old boy, May 2020.

The instructions and restrictions mentioned include, clockwise from the upper left-hand corner: “keep distance”; “stay home”; “don’t greet with hands’; “wash your hands”; “wear a mask”; and “don’t travel.”

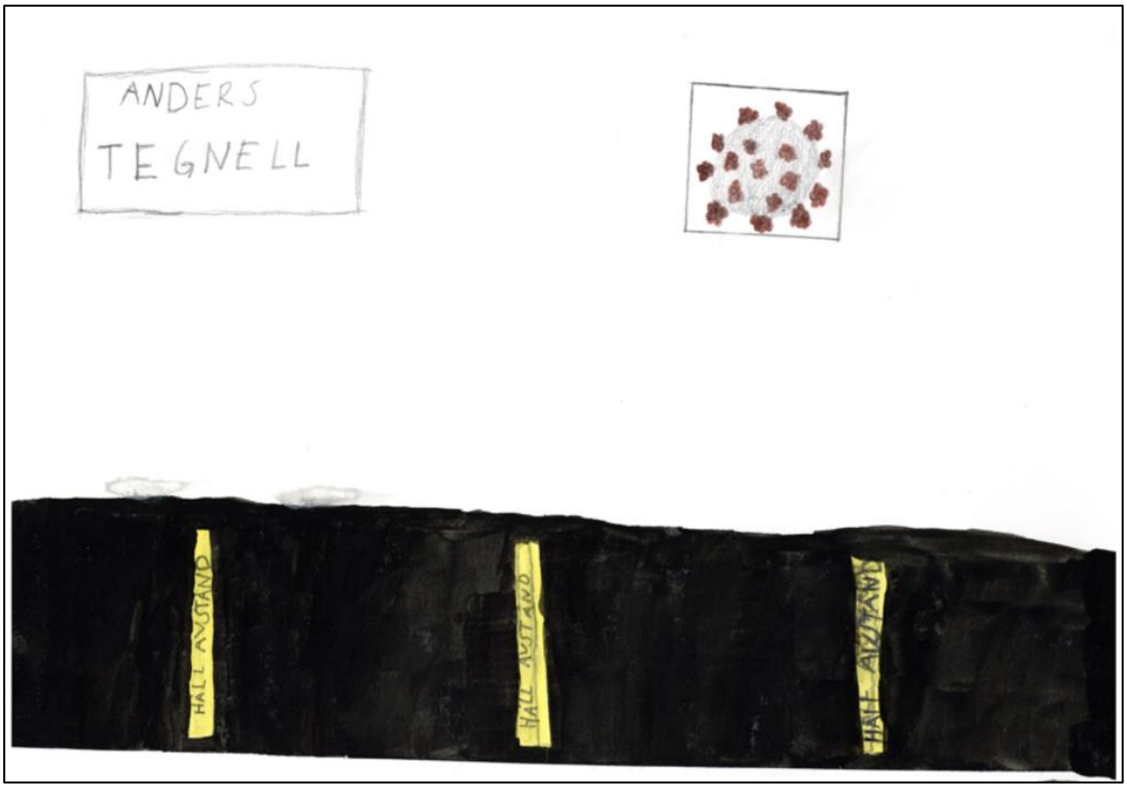

Drawing 8 (no. 215): 13-year-old boy. May 2020.

Anders Tegnell is named, and the yellow markings on the floor read “keep distance”.

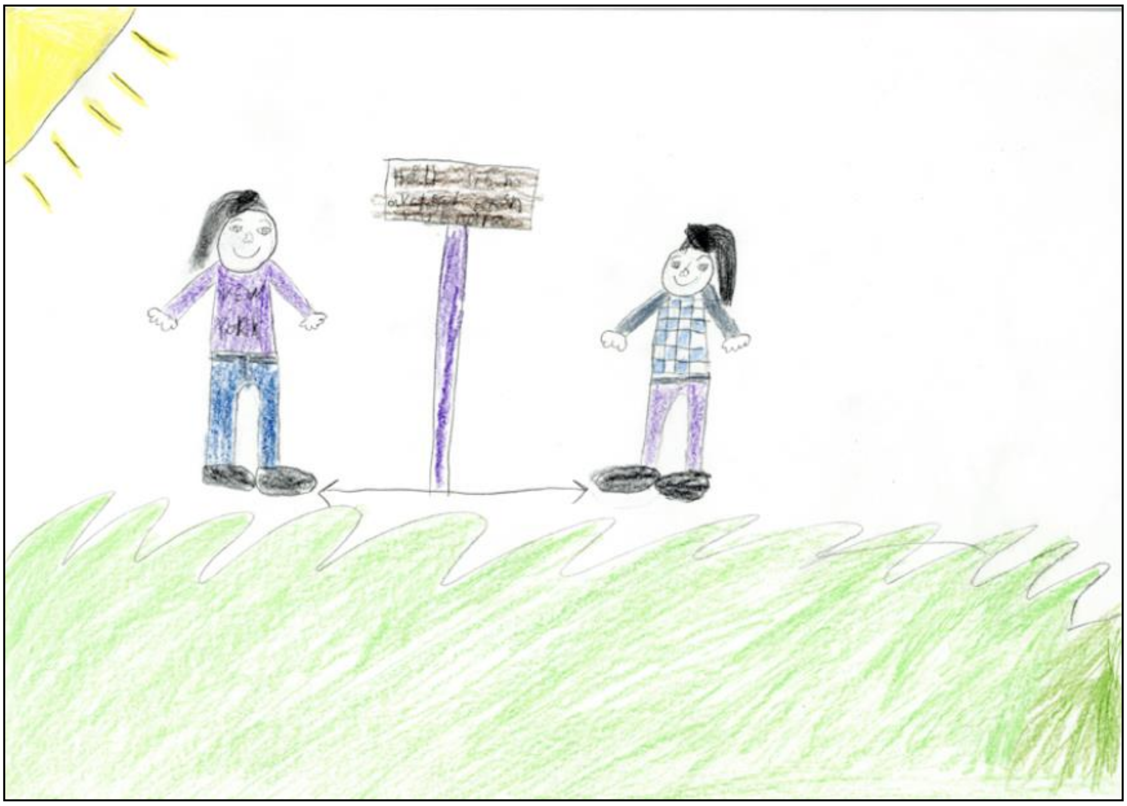

Drawing 9 (no. 758). 14-year-old girl, June 2020.

The text on the sign reads: “keep one meter distance from each other”, (“hall 1,5m avstånd från varandra”).

Motif 2: Depictions of the coronavirus

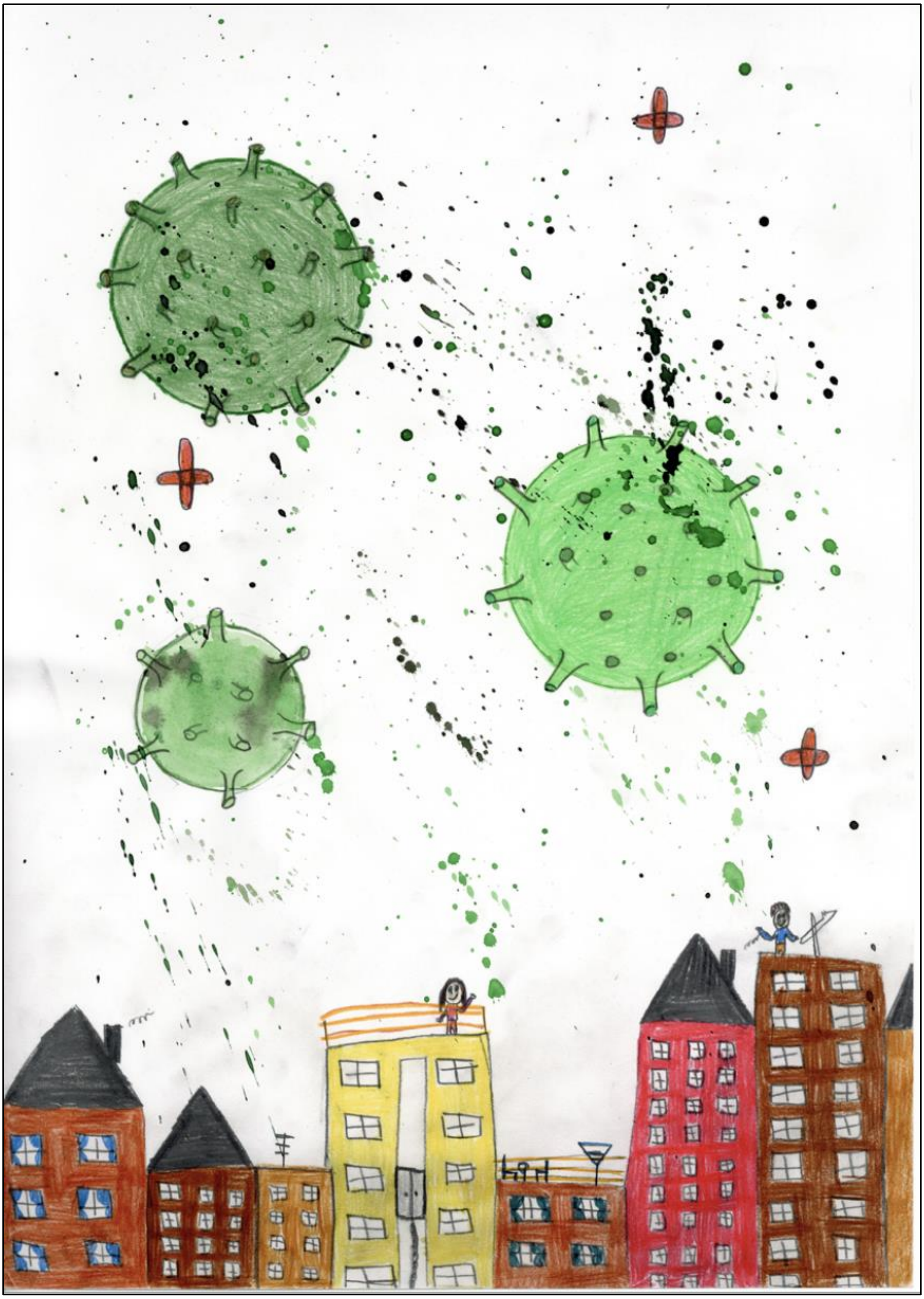

Drawing 10 (no. 896): 14-year-old girl. May 2020.

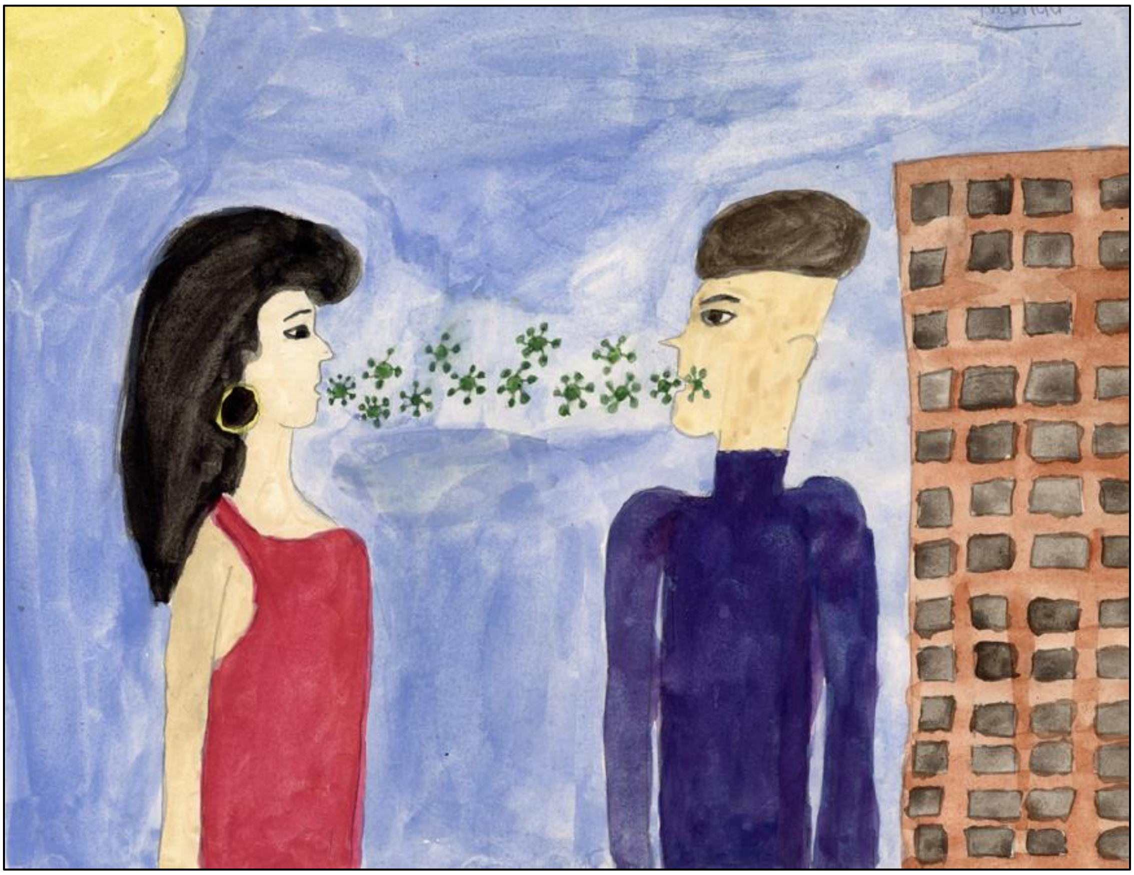

Drawing 11 (no. 411). 14-year-old girl, May 2020.

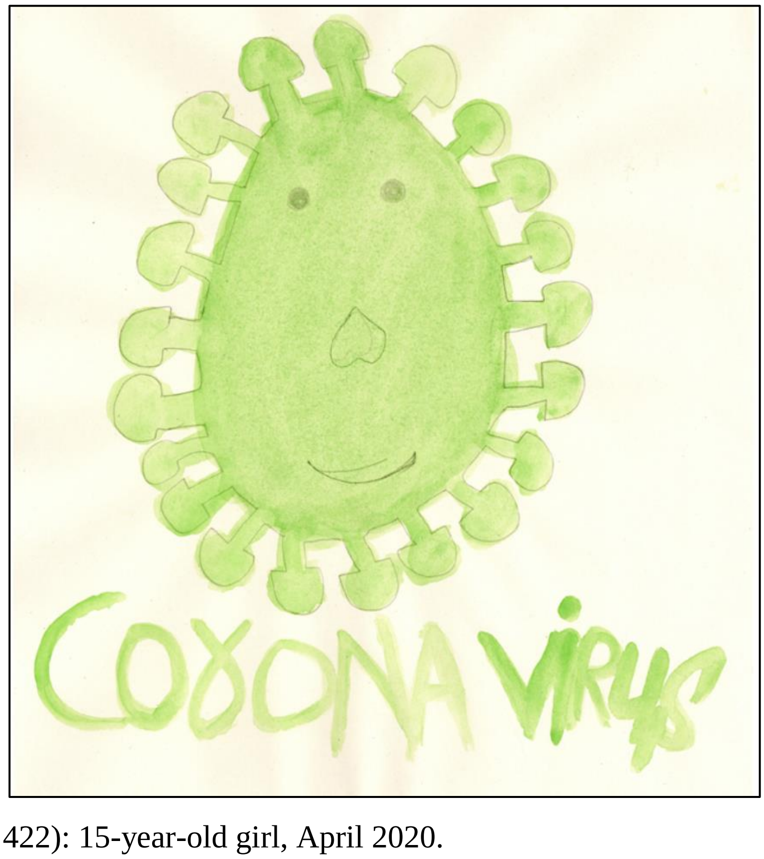

Drawing 12 (no. 422): 15-year-old girl, April 2020.

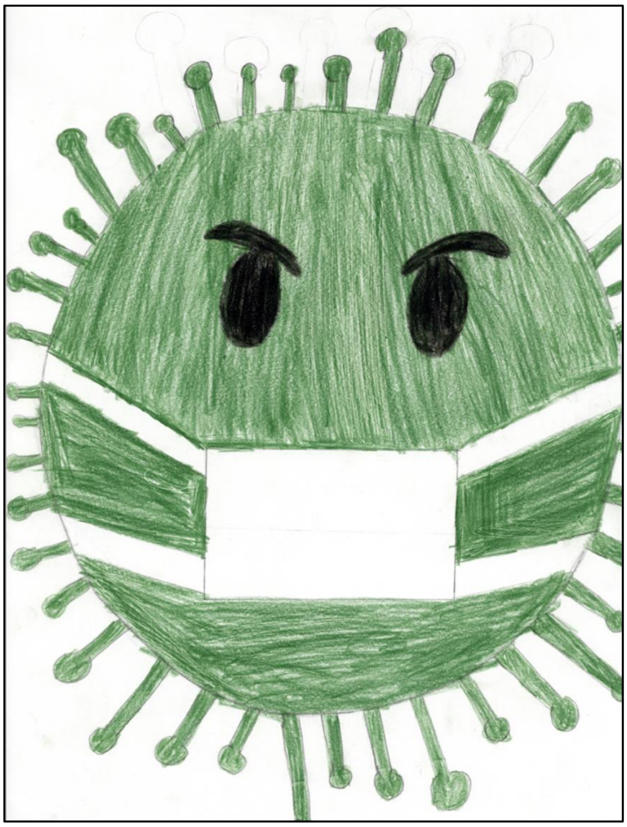

Drawing 13 (no. 905). 13-year-old girl, May 2020.

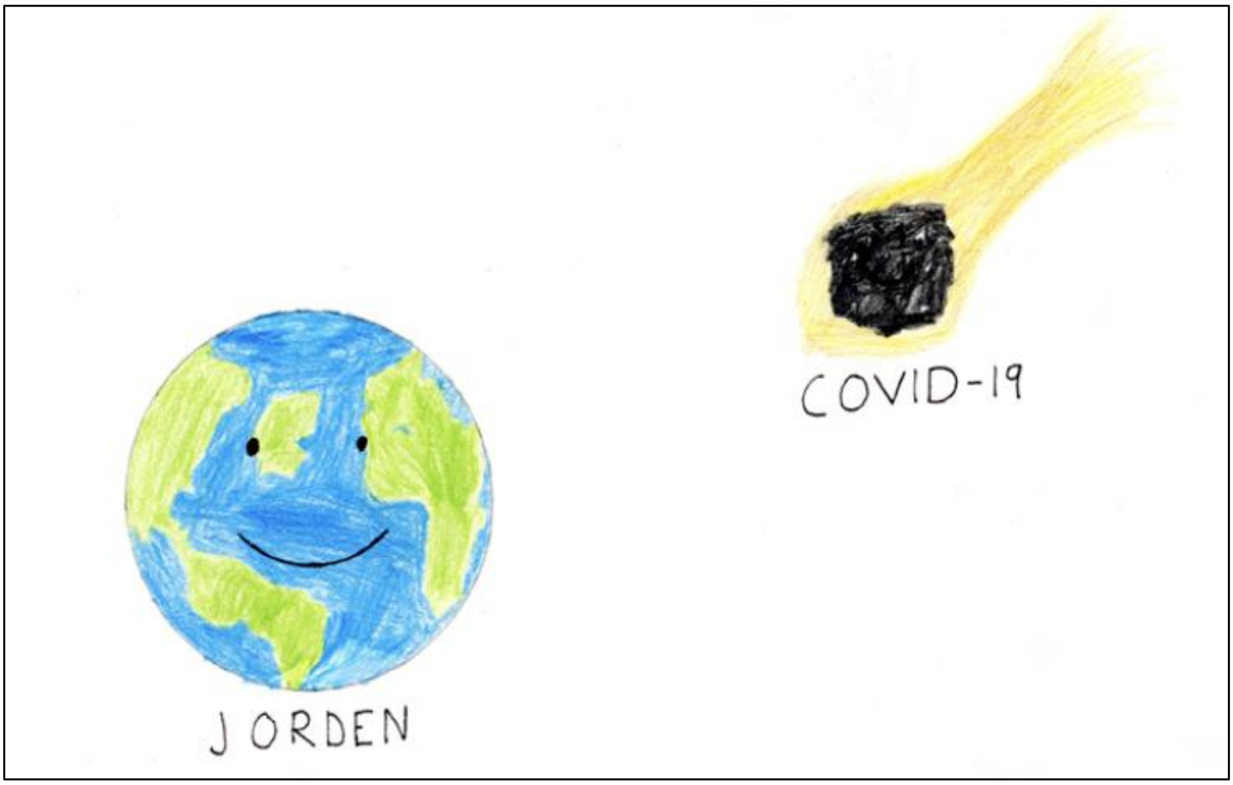

Drawing 14 (no. 662). 13-year-old boy, June 2020.

Jorden means the earth.

Motif 3: People

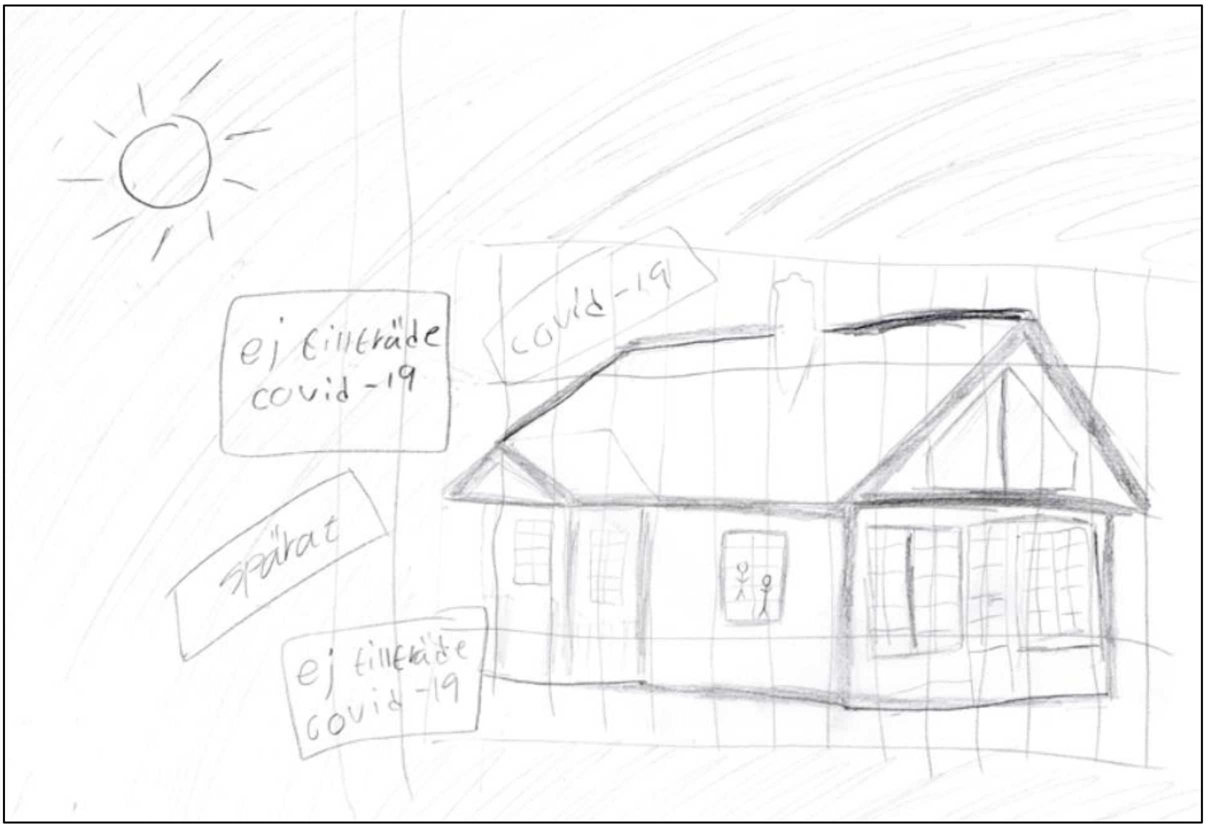

Drawing 15 (no. 26). 13-year-old girl, April 2020.

The Swedish text reads “no entrance COVID-19,” and “blocked”.

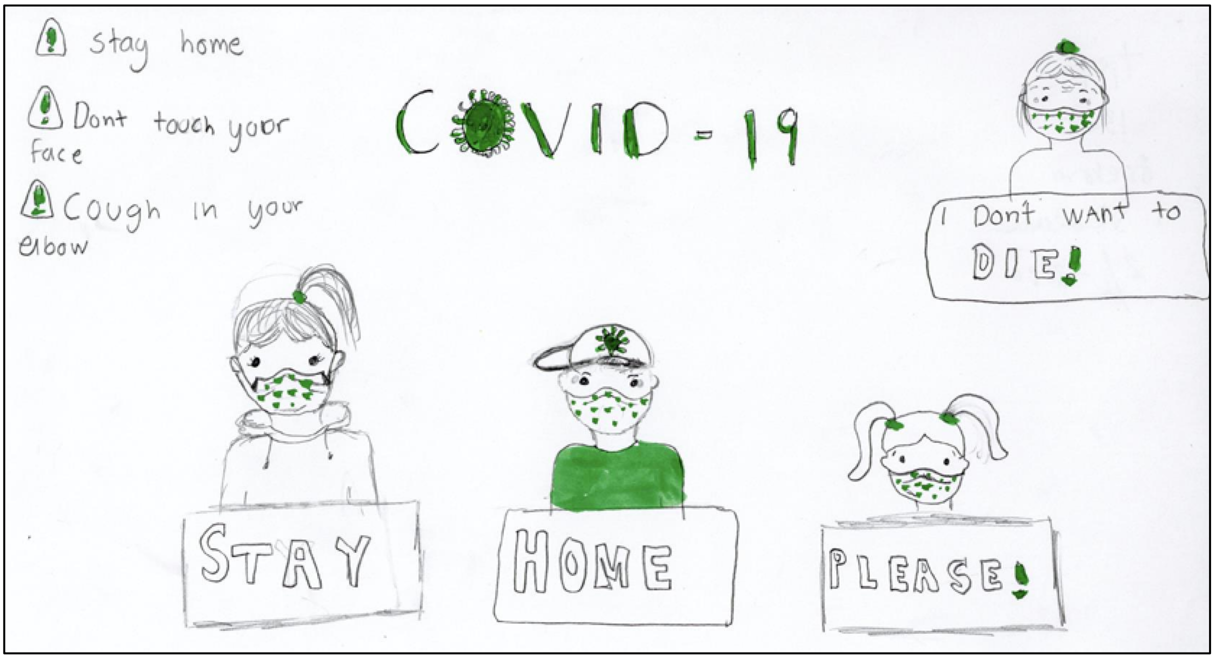

Drawing 16 (no. 787). 13-year-old girl, June 2020.

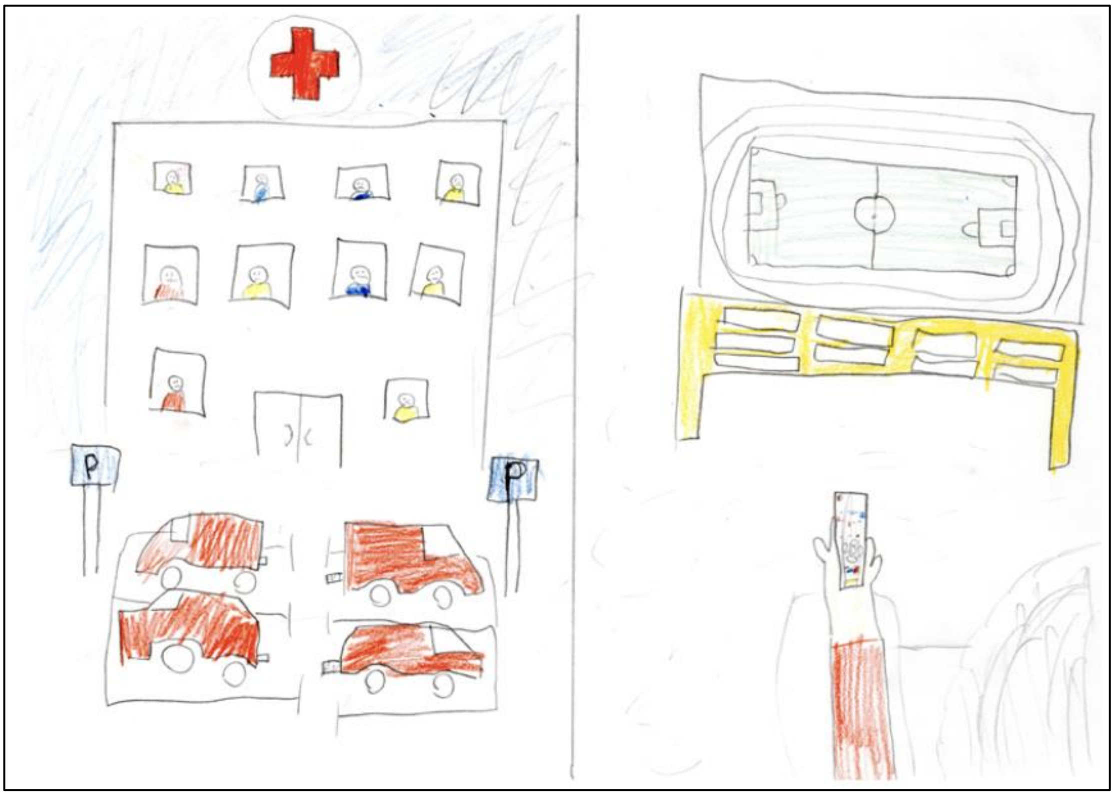

Drawing 17 (no. 688). 14-year-old boy, June 2020.

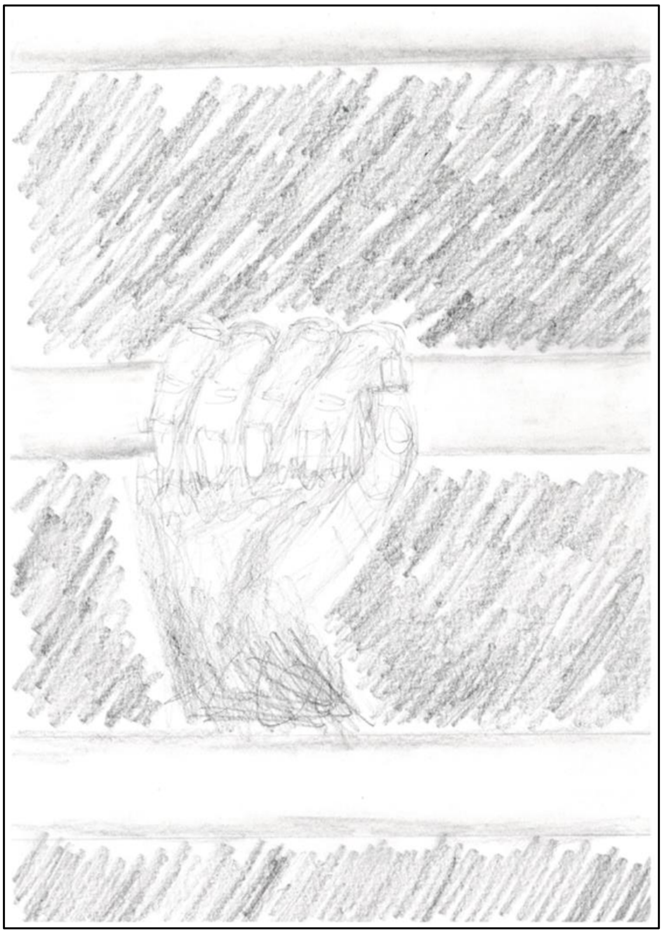

Drawing 18 (no. 685). 15-year-old girl, June 2020.

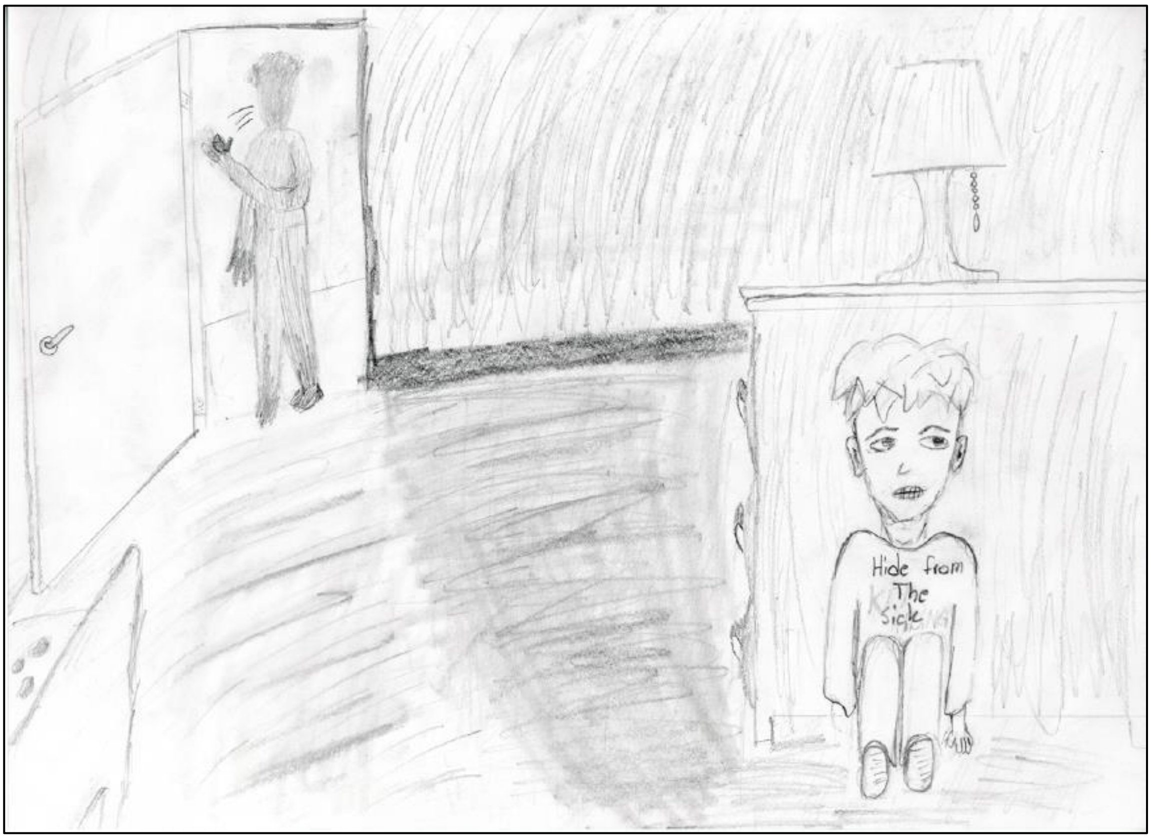

Drawing 19 (no. 916). 13-year-old boy, May 2020.

The child’s t-shirt reads “hide from the sick,” written in English.

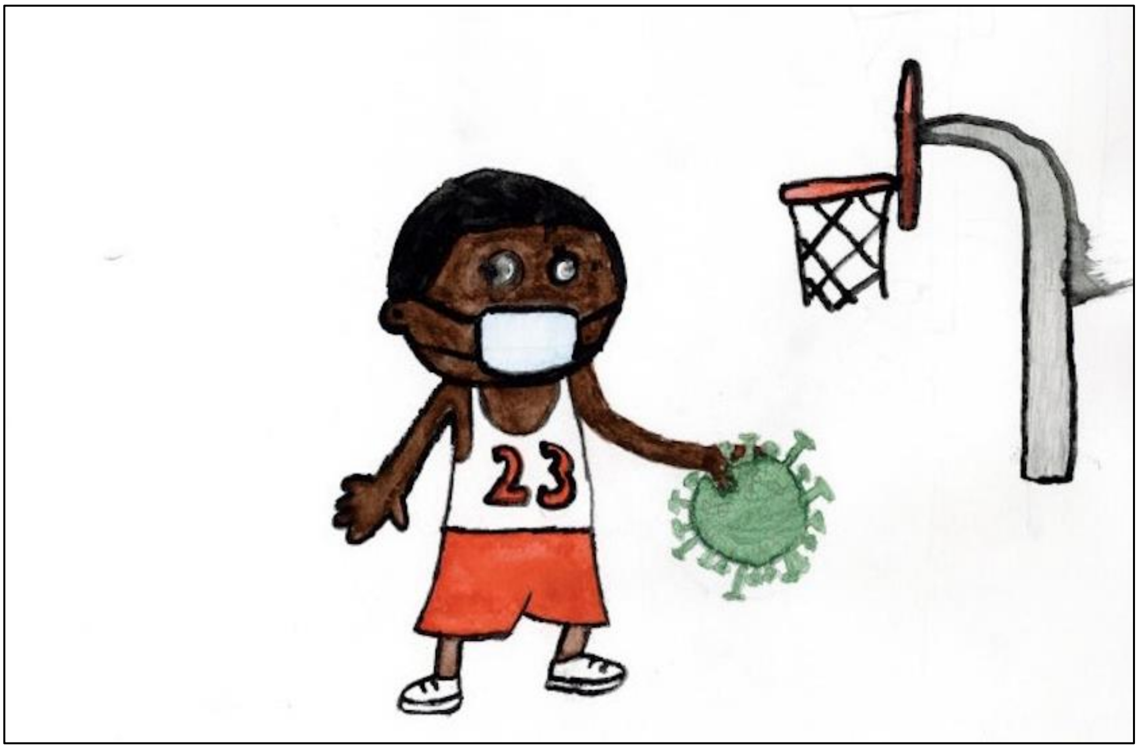

Drawing 20 (no. 638). 13-year-old girl, May 2020.

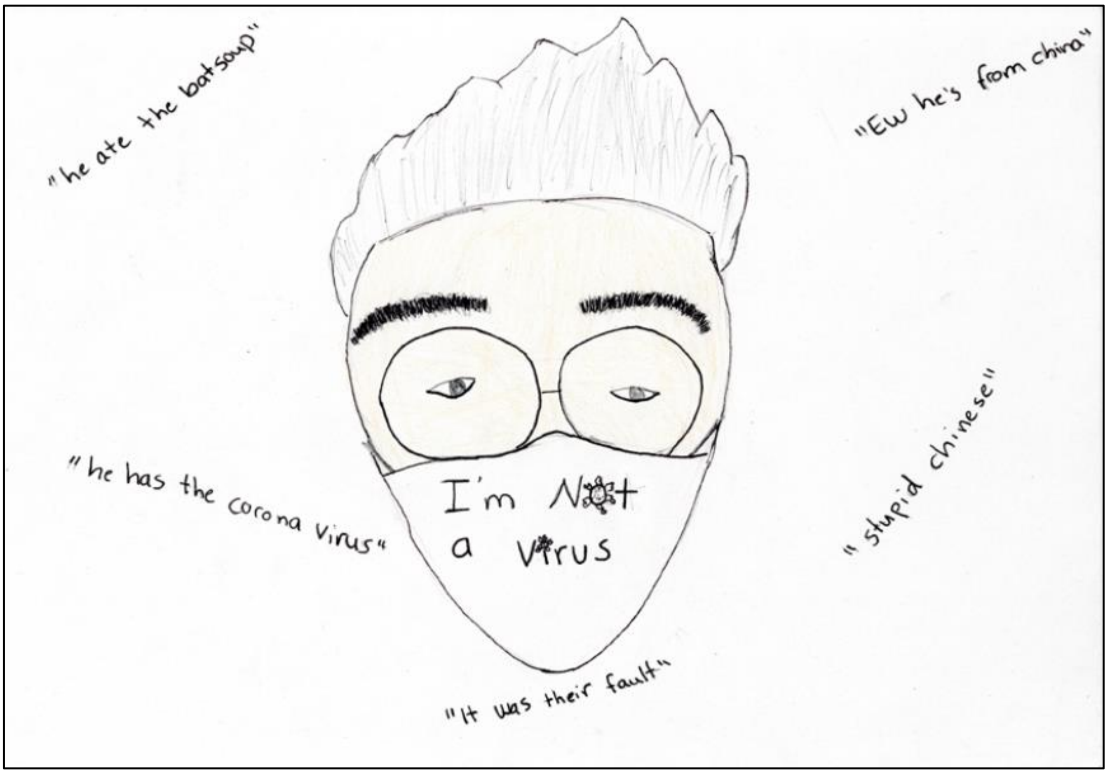

Drawing 21 (no. 778). 14-year-old girl, June 2020.

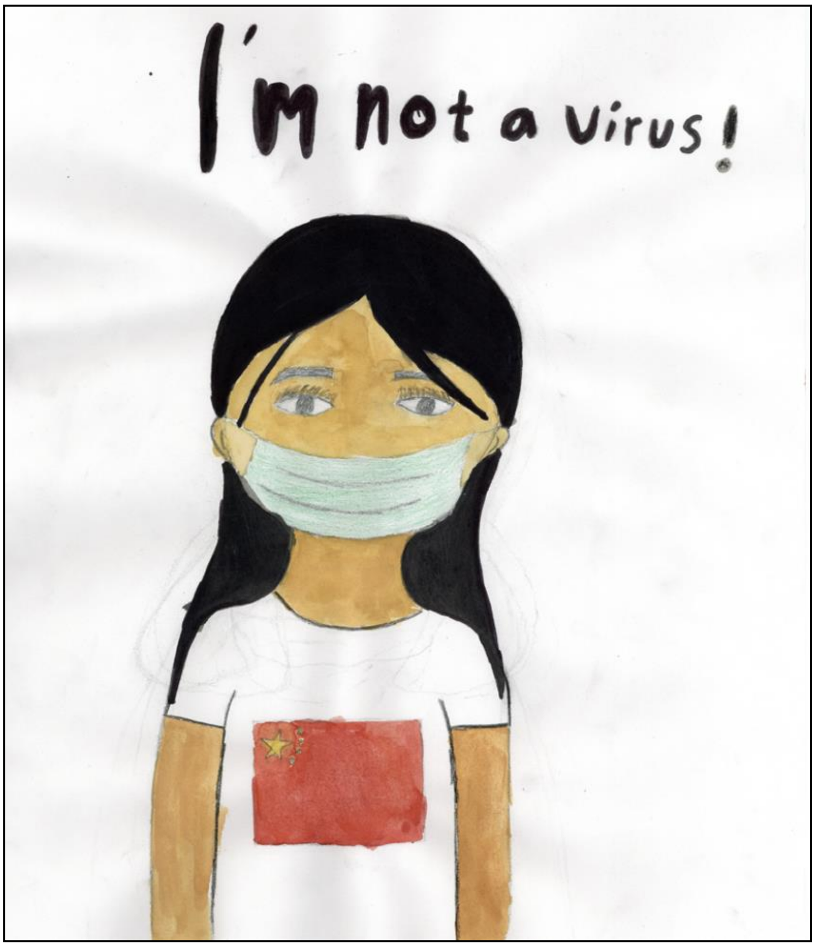

Drawing 22 (no. 899). 13-year-old girl, May 2020.

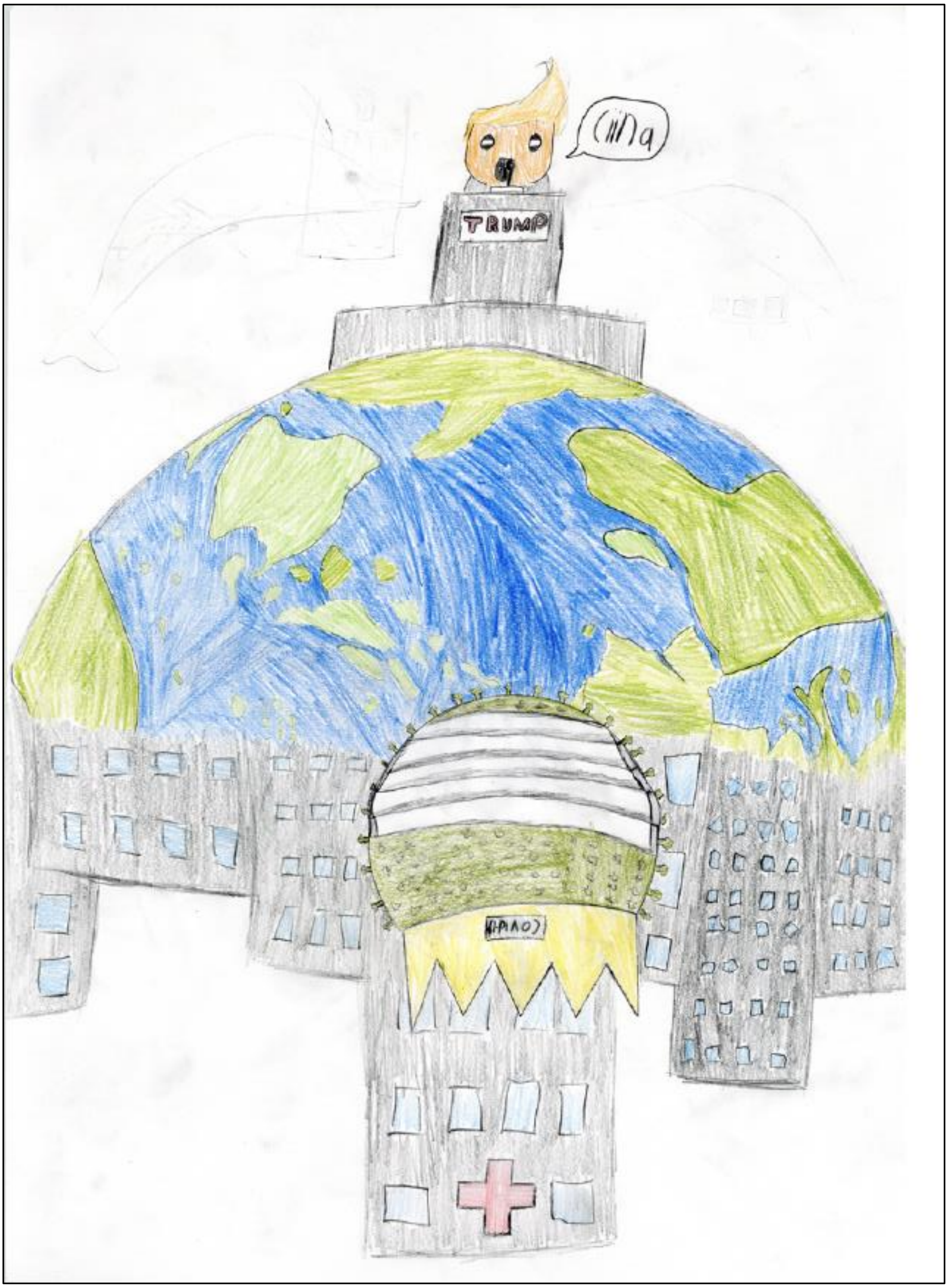

Drawing 23 (no. 875). 13-year-old boy, May 2020.

Trump seems to be saying “Ciina”, likely a misspelling of “Kina” or “China”.

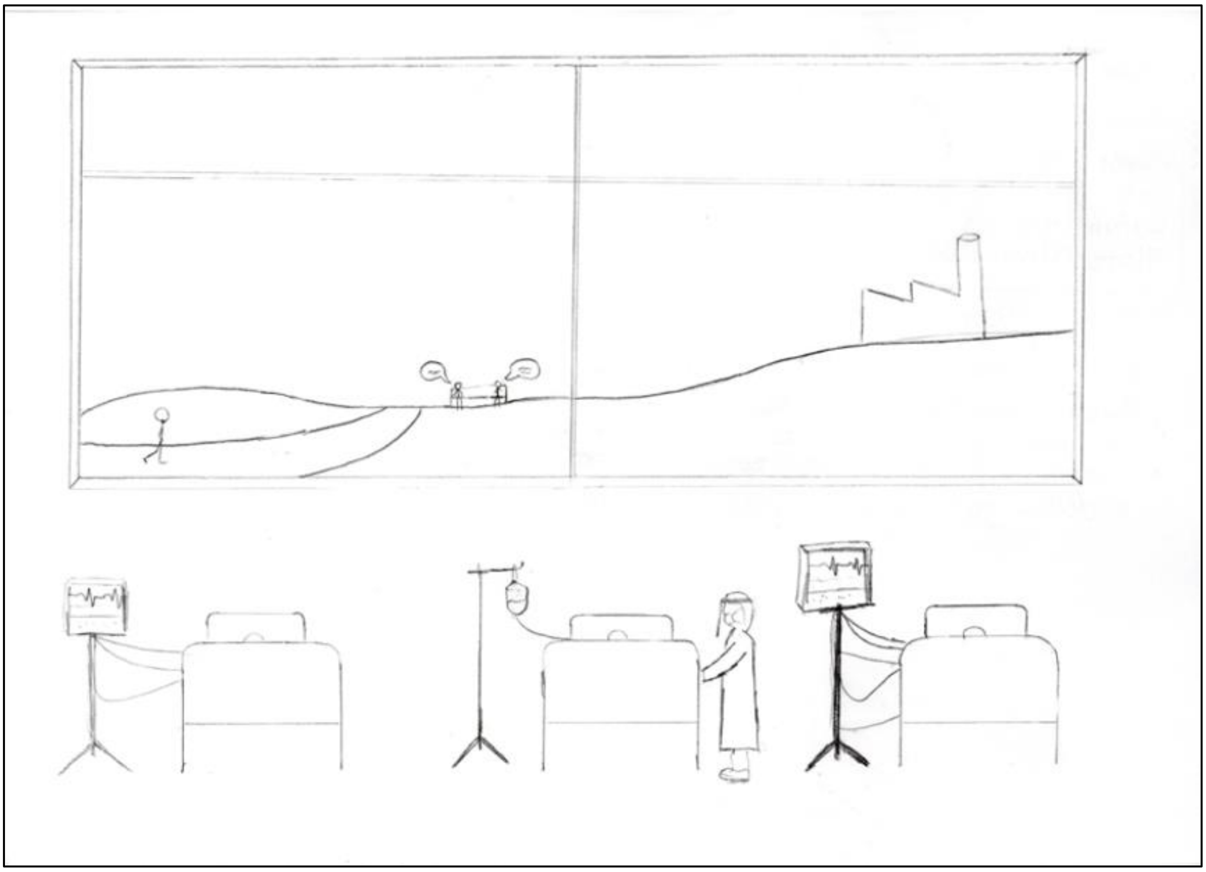

Drawing 24 (no. 728). 15-year-old girl, June 2020.

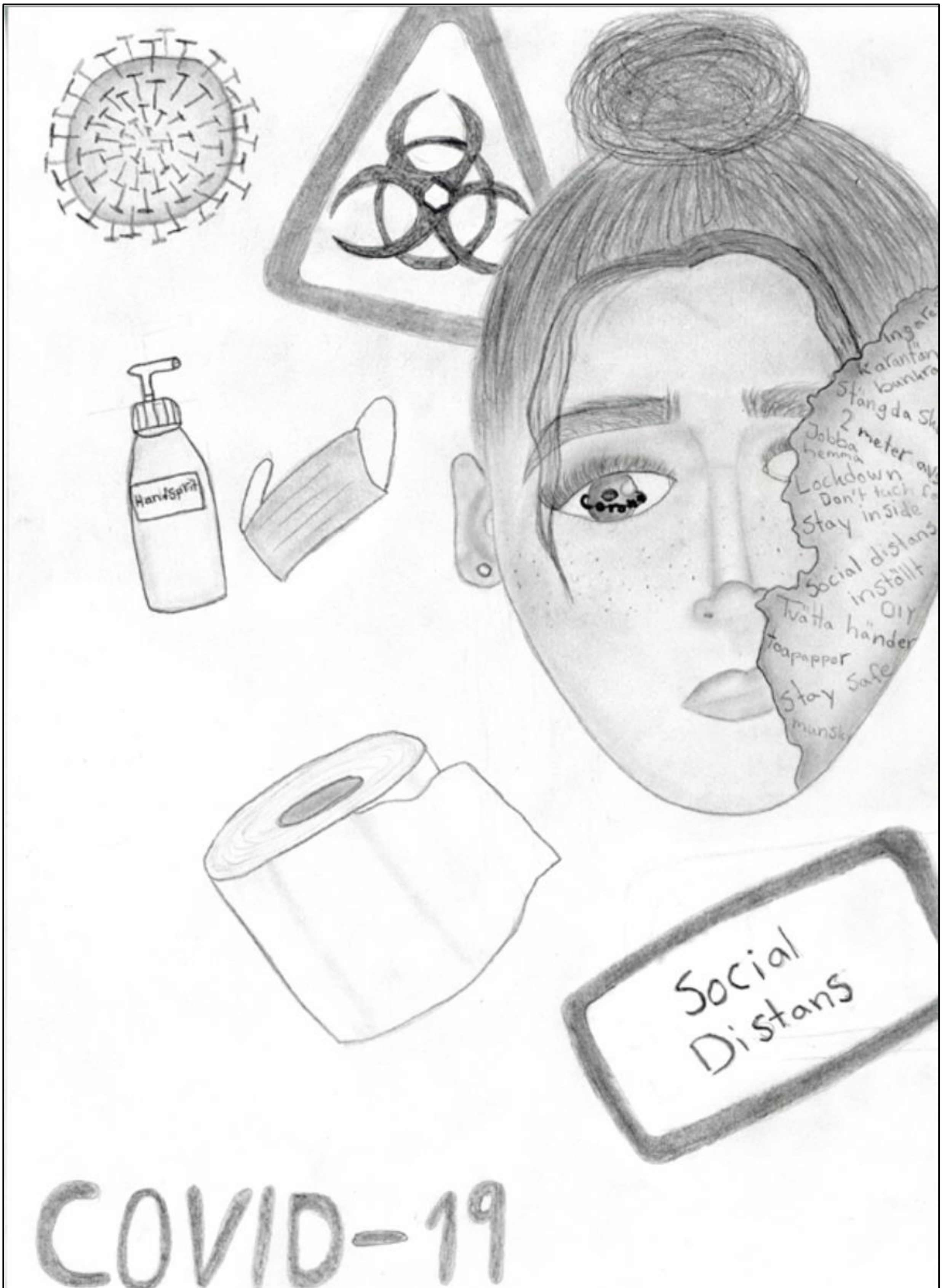

Drawing 25 (no. 696). 14-year-old girl, May 2020.

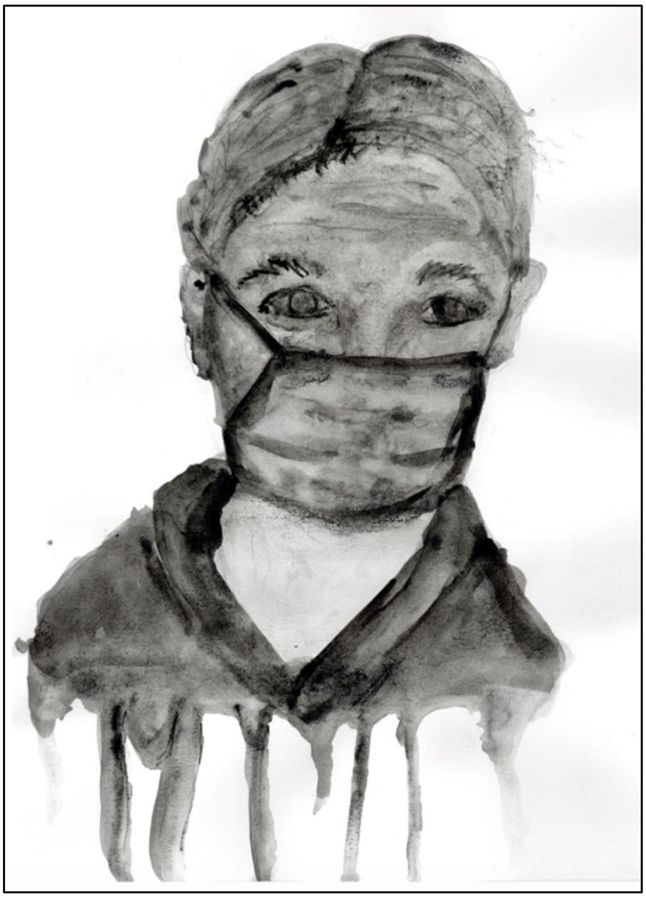

Drawing 26 (no. 210). 13-year-old girl, June 2020.

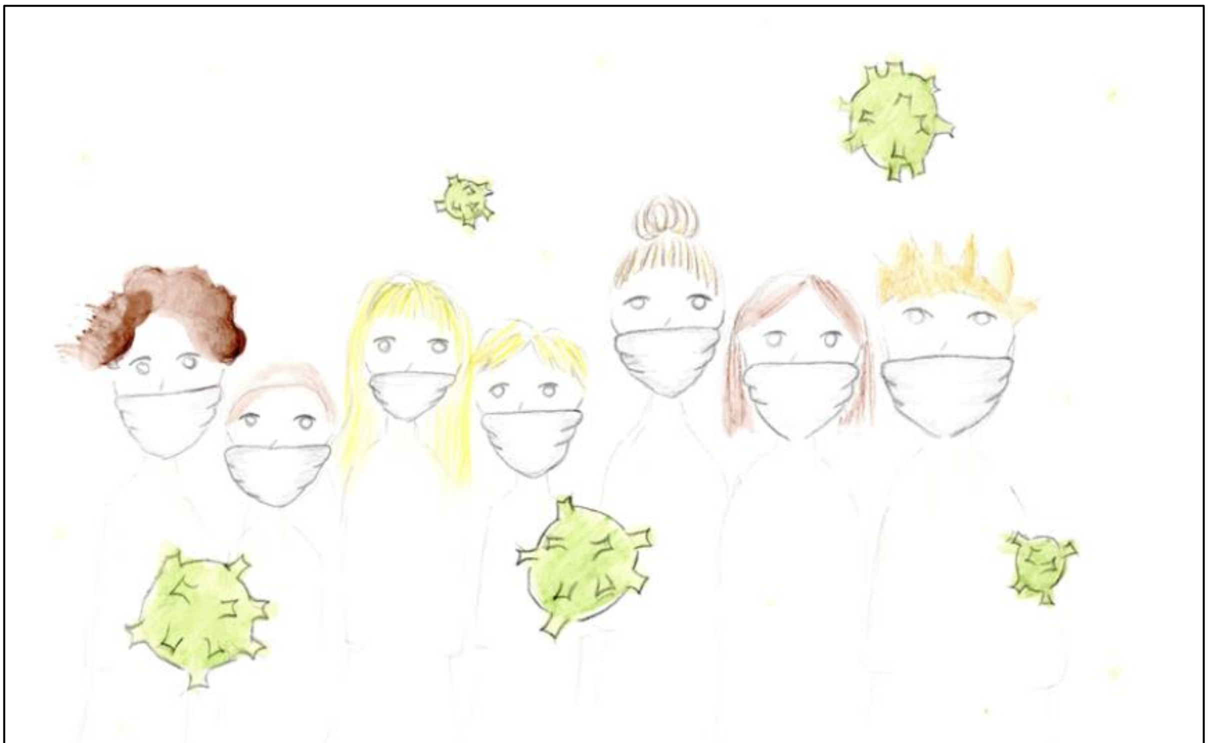

Drawing 27 (no. 652). 13-year-old girl, May 2020.

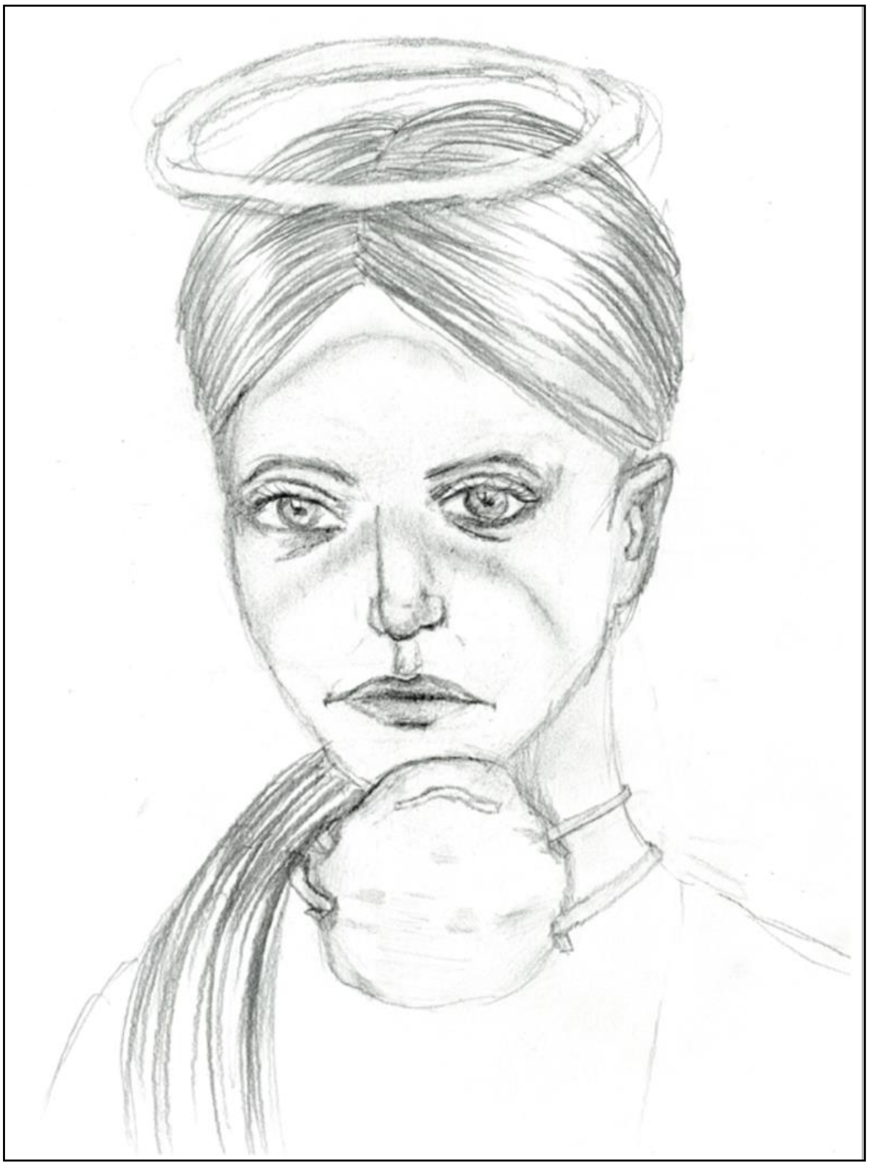

Drawing 28 (no. 683). 14-year-old girl, June 2020.

Motif 4: Places

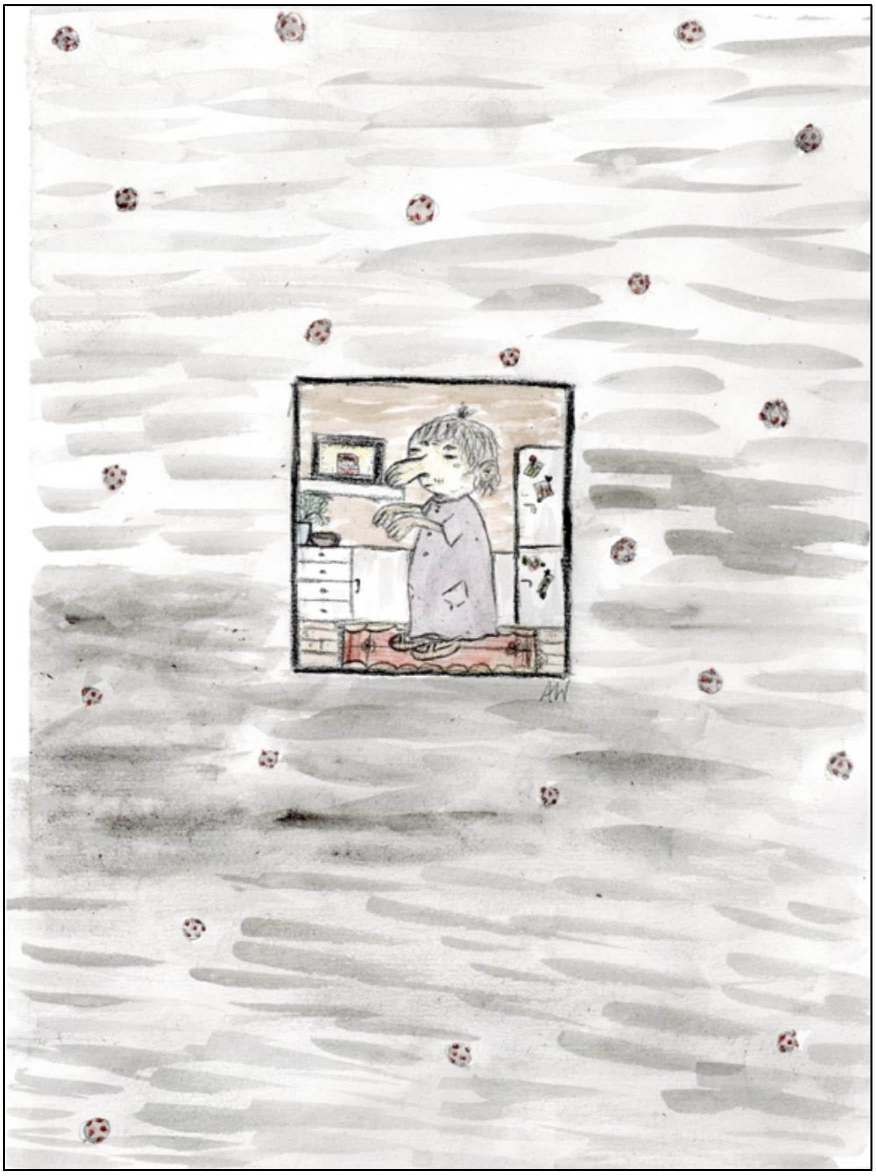

Drawing 29 (no. 218). 13-year-old girl, May 2020.

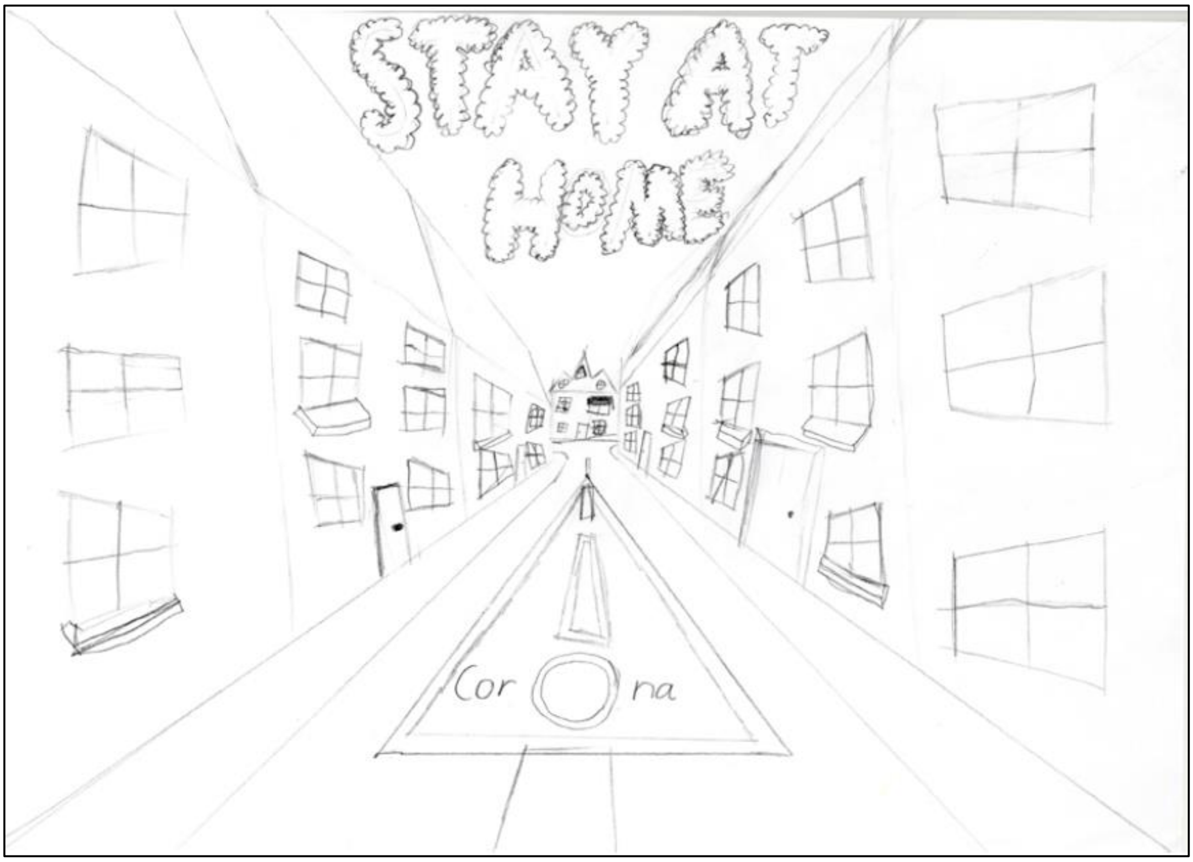

Drawing 30 (no. 695). 13-year-old boy, June 2020.

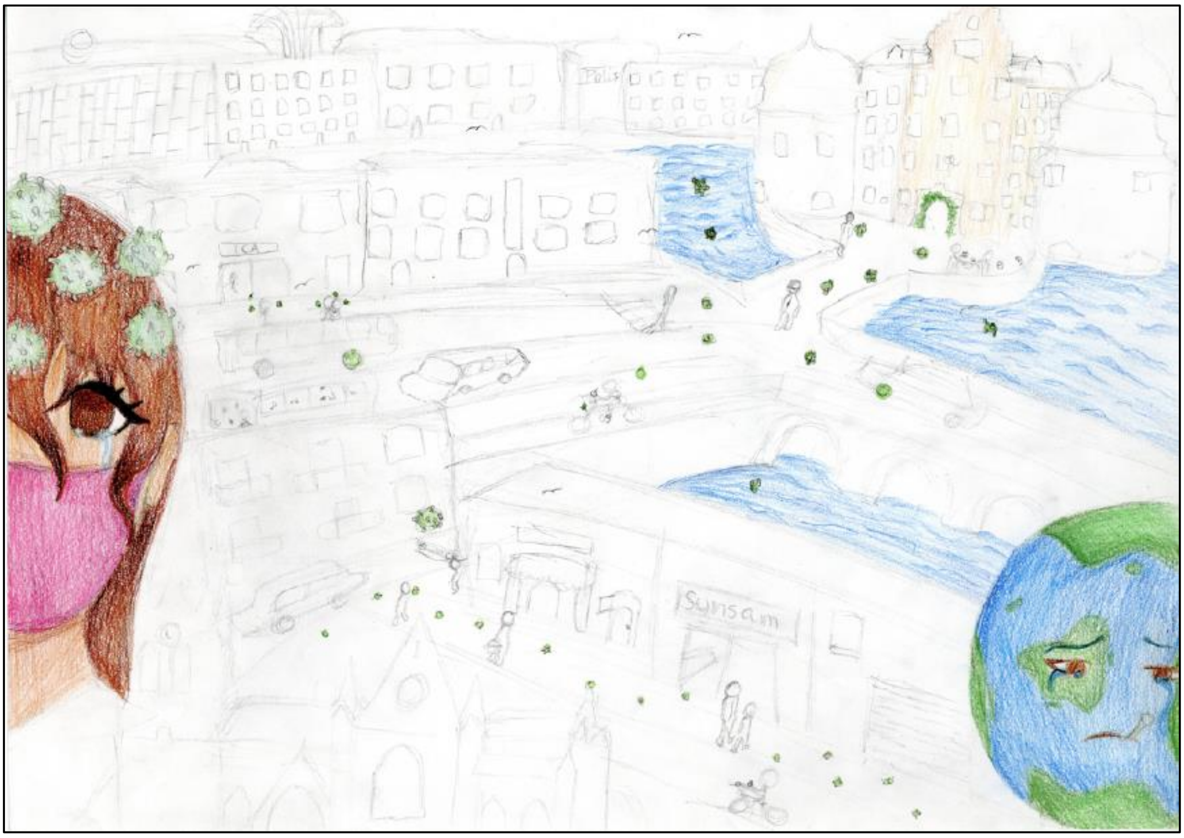

Drawing 31 (no. 868). 13-year-old girl, May 2020.

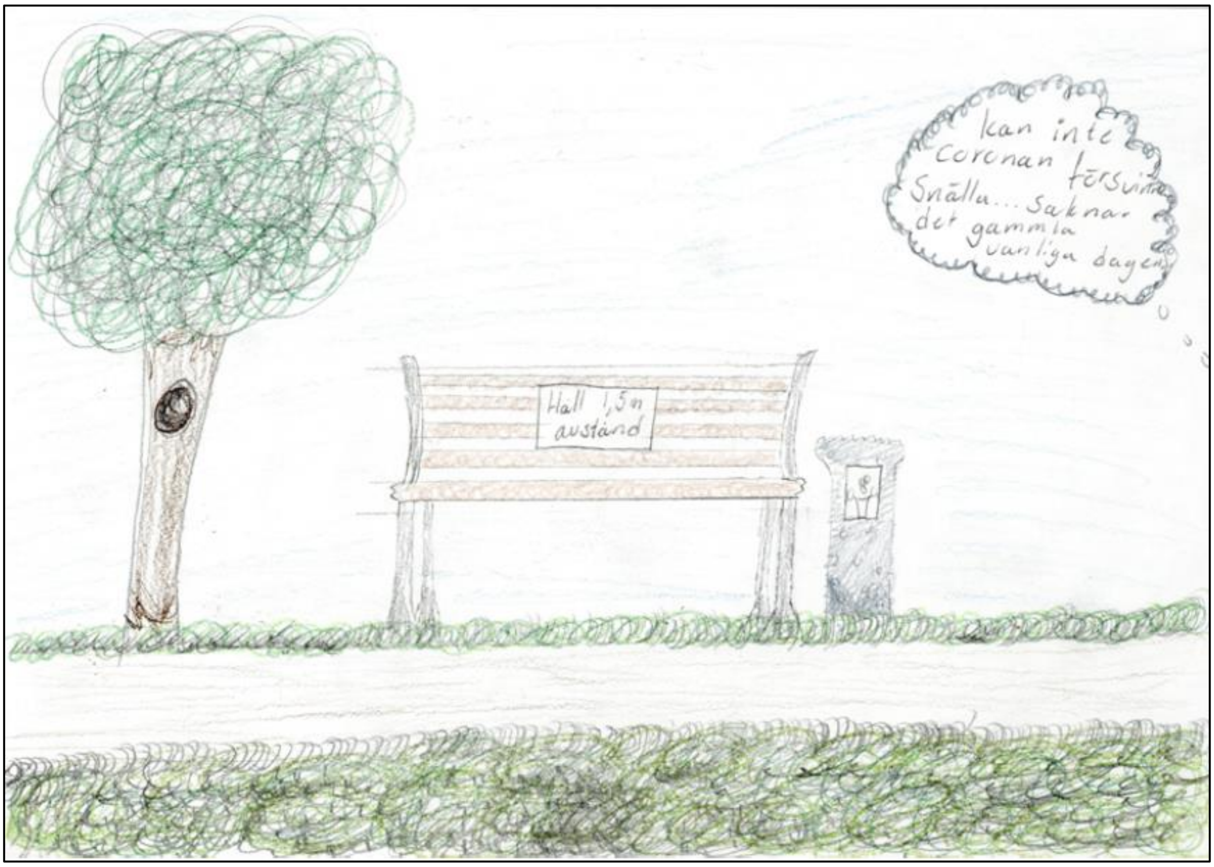

Drawing 32 (no. 756). 14-year-old girl, June 2020.

The text on the bench reads “keep 1,5m distance,” and the speech bubble states, “can Corona please disappear…I miss the old usual day”.

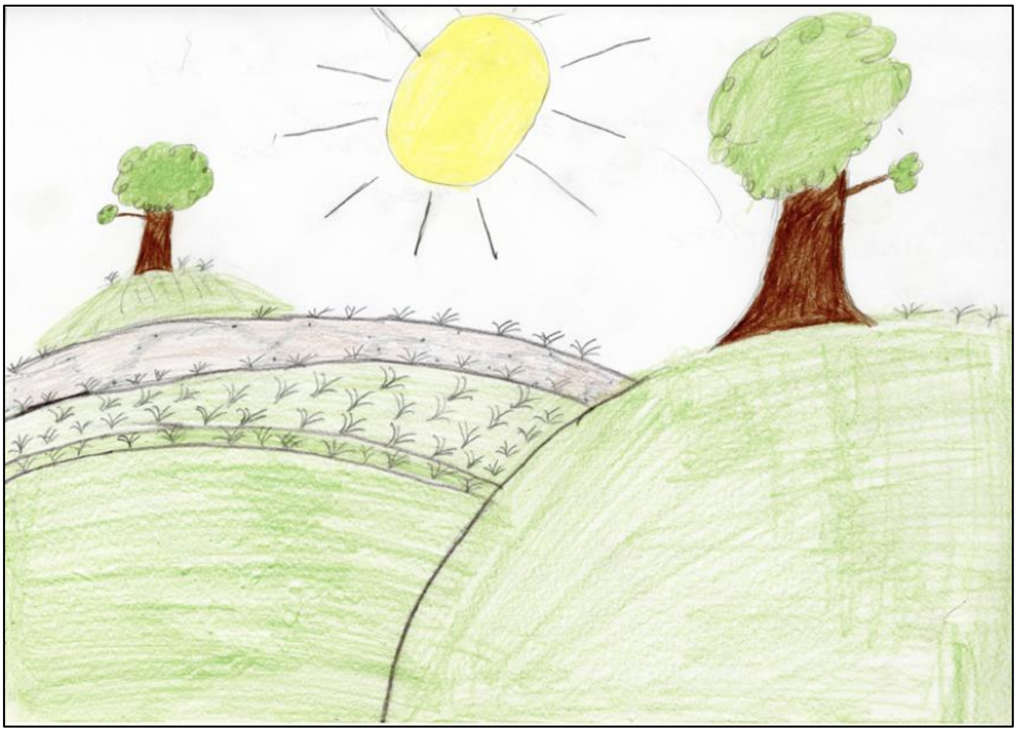

Drawing 33 (no. 748). 14-year-old boy, June 2020.

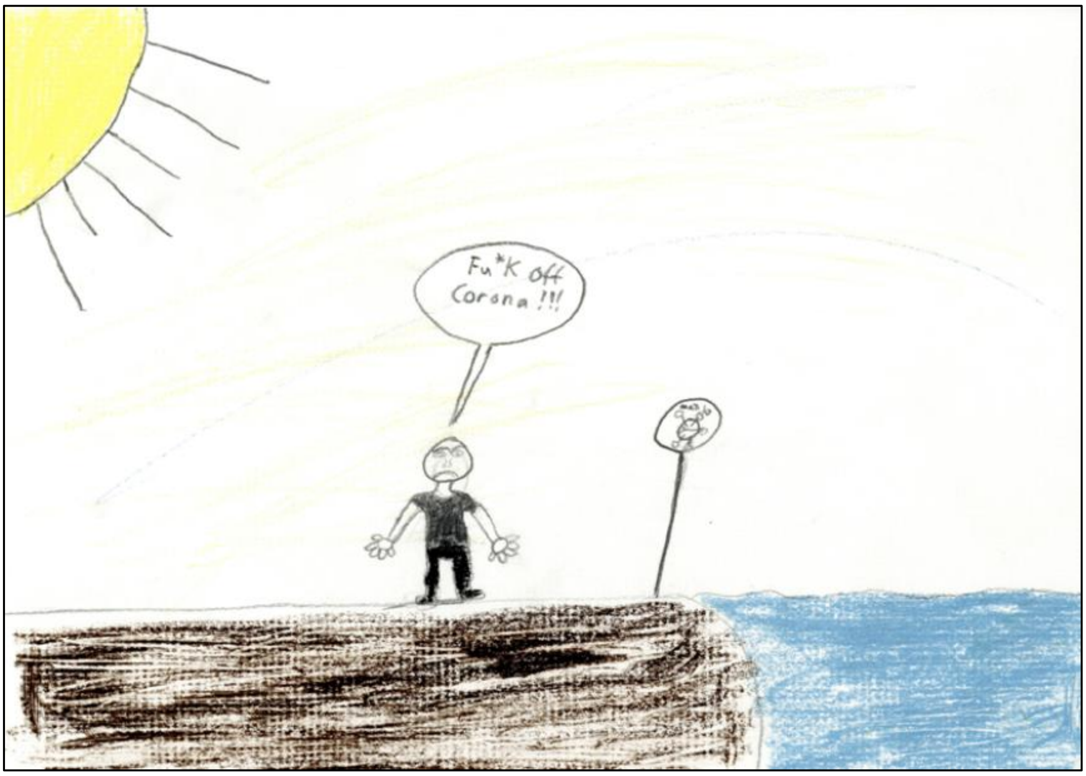

Drawing 34 (no. 742). 14-year-old boy, June 2020.

The sign shows a Coronavirus particle and the text “max 10”.

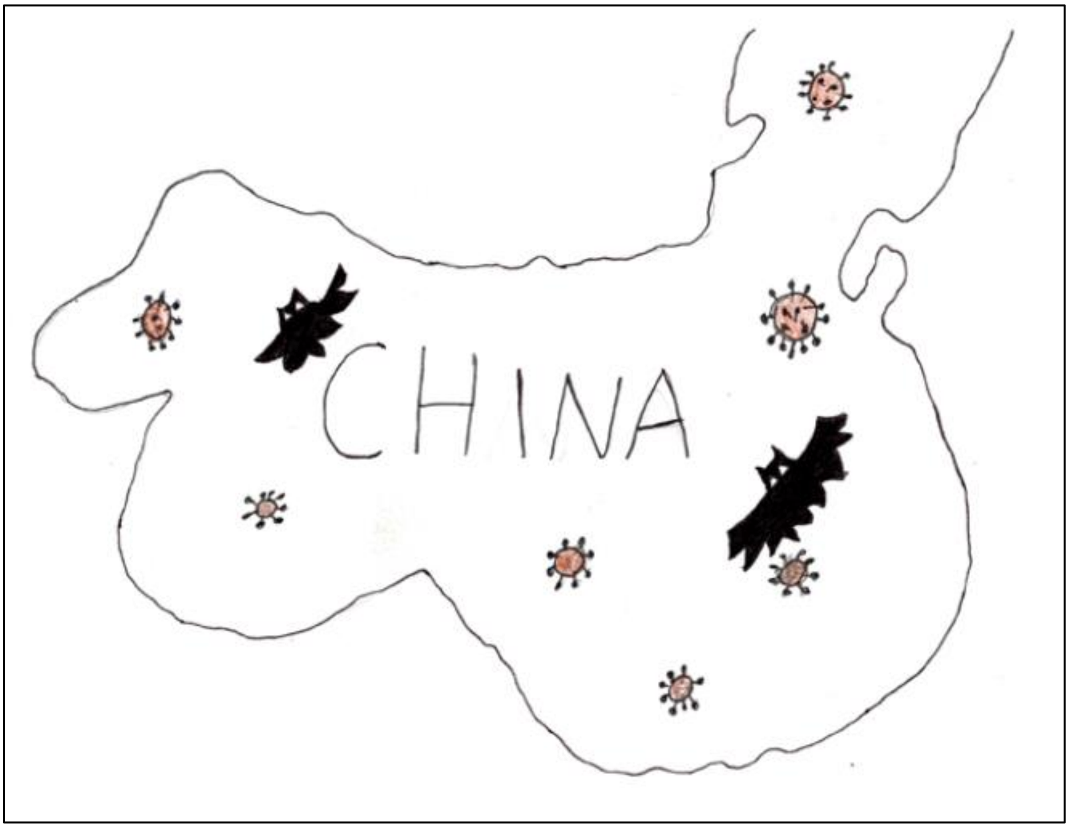

Drawing 35 (no. 712). 14-year-old girl, June 2020.

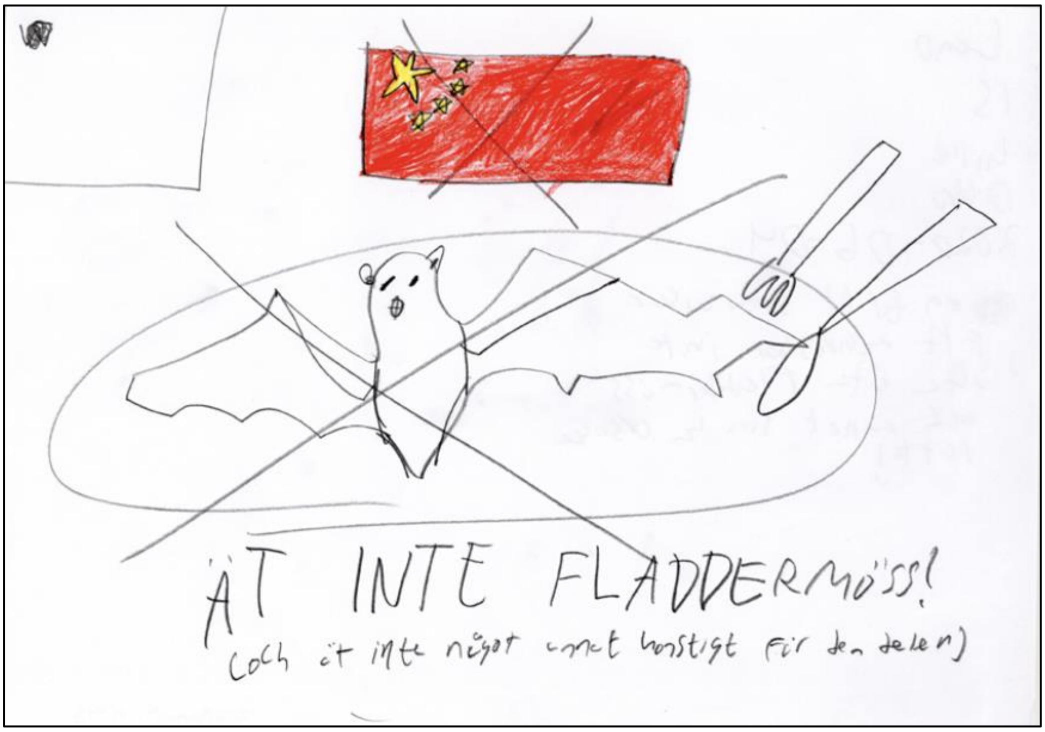

Drawing 36 (no. 743). 15-year-old boy, June 2020.

The text reads “don’t eat bats! (and don’t eat anything else strange for that matter)”.

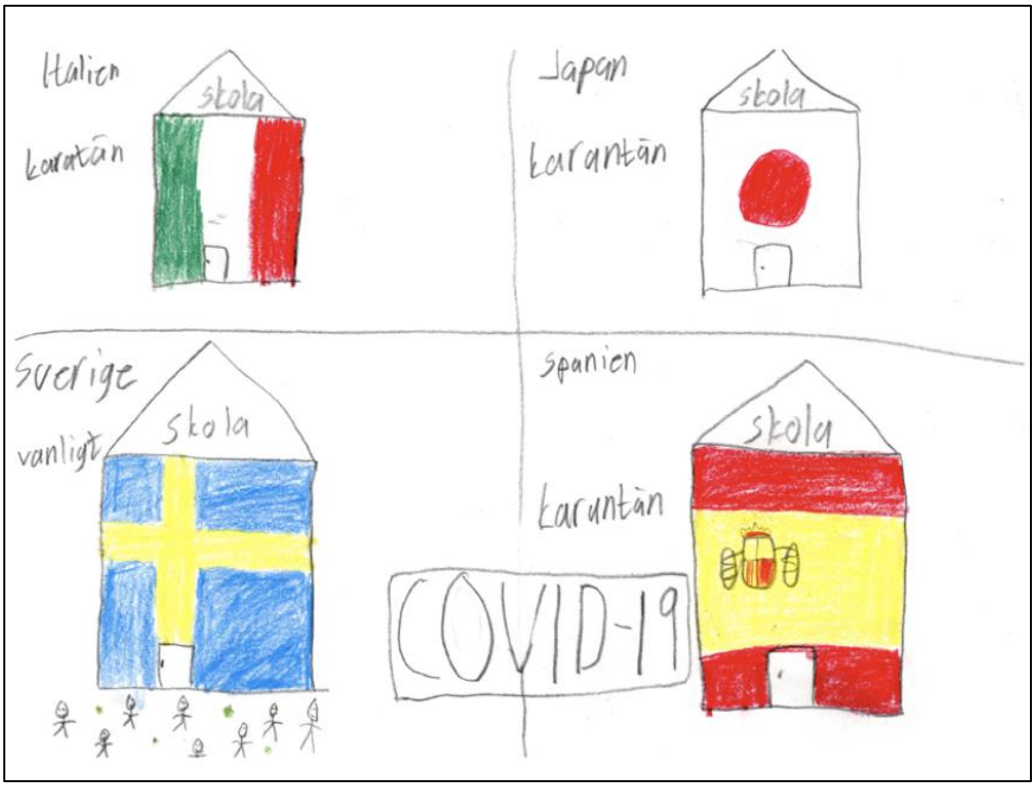

Drawing 37 (no. 674). 14-year-old boy, June 2020.

“Skola” is school, “karantän” is quarantine and “vanligt” means as usual. The countries represented are Italy, Japan, Spain and Sweden.

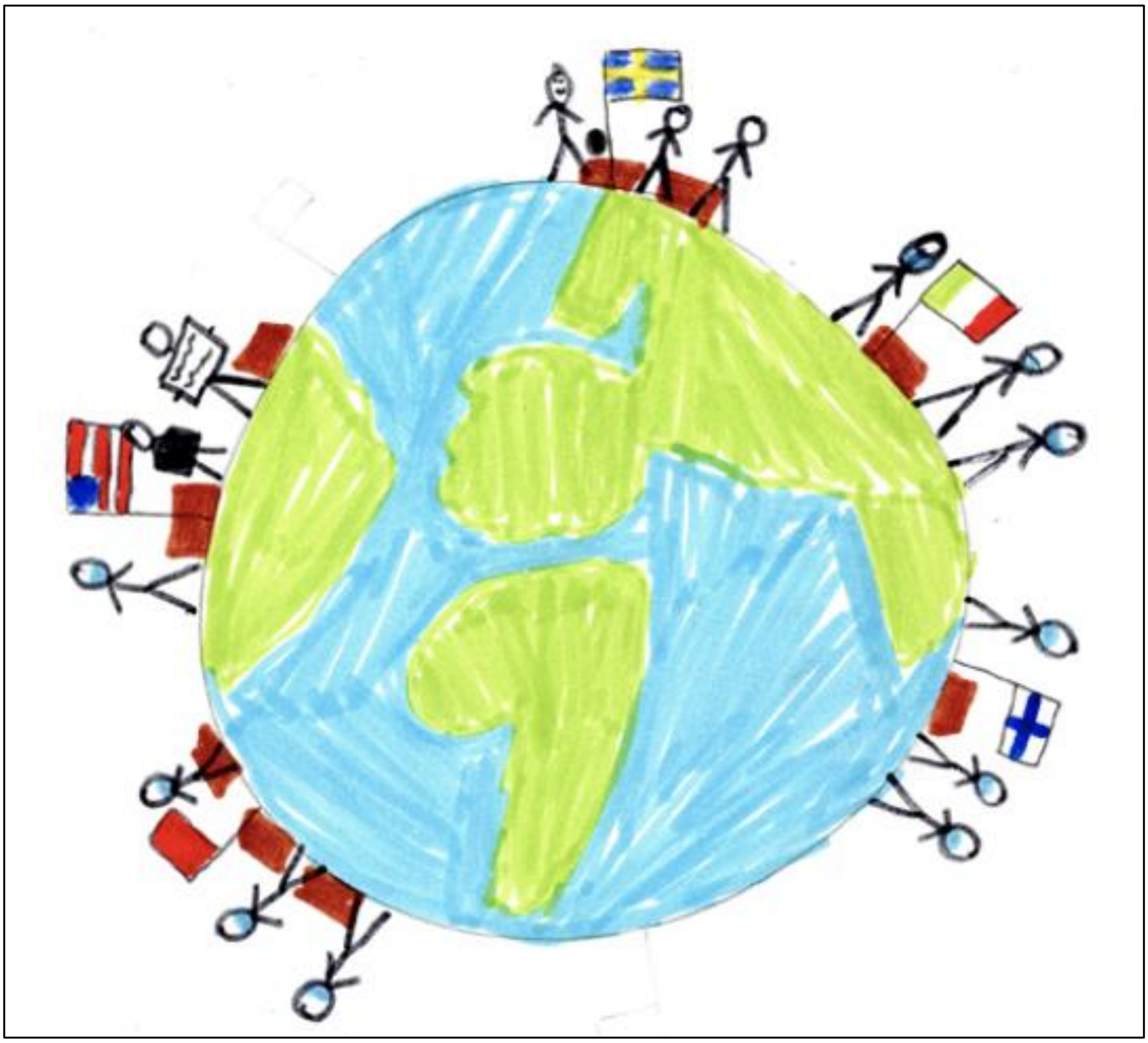

Drawing 38 (no. 679). 15-year-old girl, June 2020.

Moving clockwise from the top of the earth, the flags look to represent Sweden, Italy, Finland, China and USA.

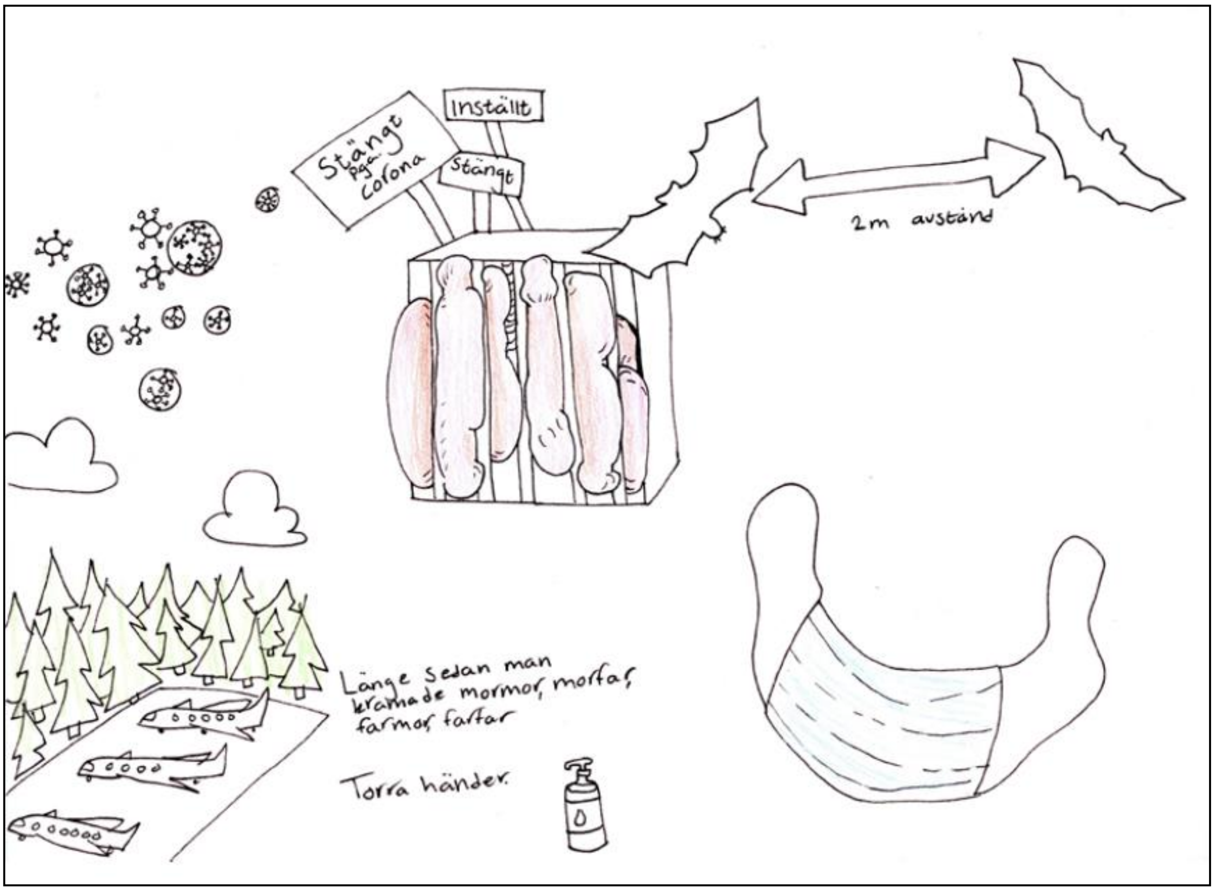

Drawing 39 (no. 715). 15-year-old girl, June 2020.

The text above on the signs reads “cancelled” (“inställt”); “closed” (“stängt”); and “closed because of corona” (“stängt pga corona”). The text between the bats says “2m distance”. Finally, the text to the right of the airplanes says, “A long time since one hugged one’s grandparents” (“Länge sedan…”), and below this, “dry hands” (“torra händer”).

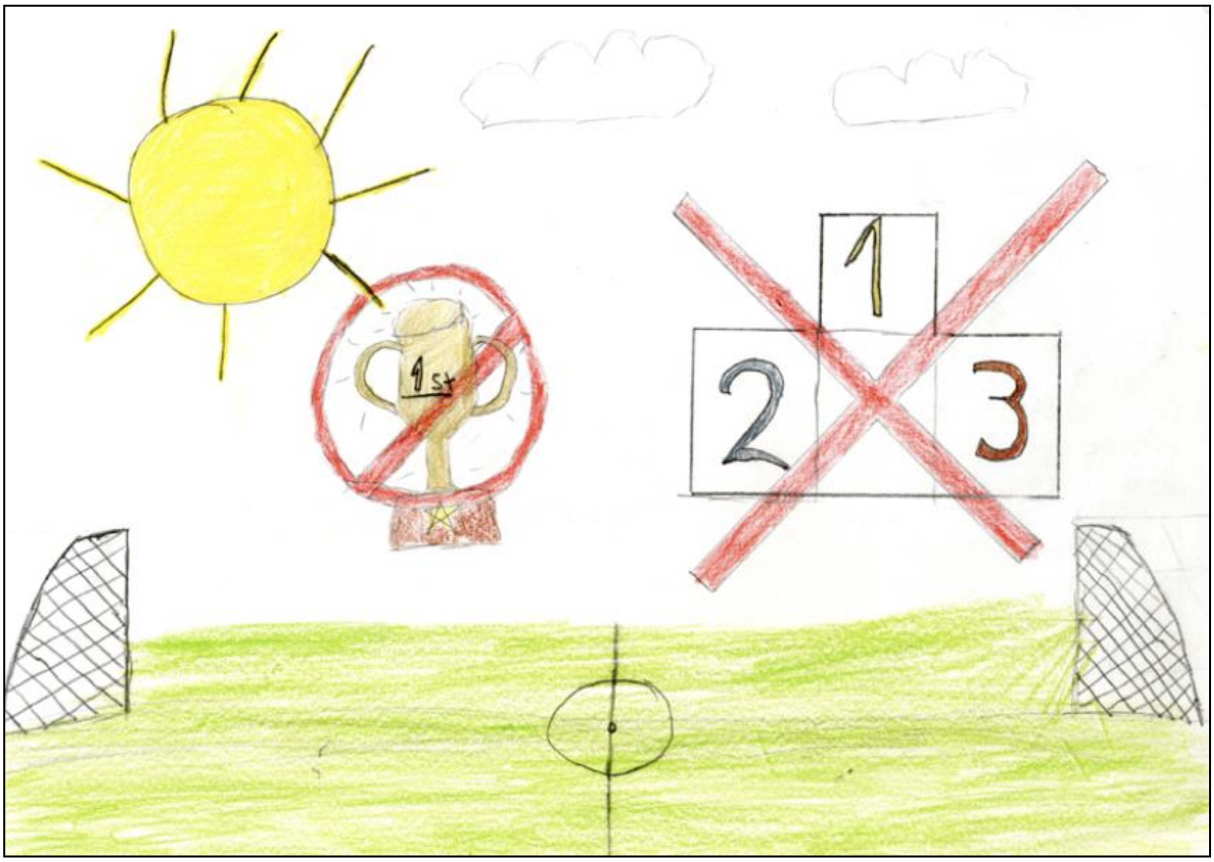

Drawing 40 (no. 691). 15-year-old boy, June 2020.

Motif 5: Depictions of the earth

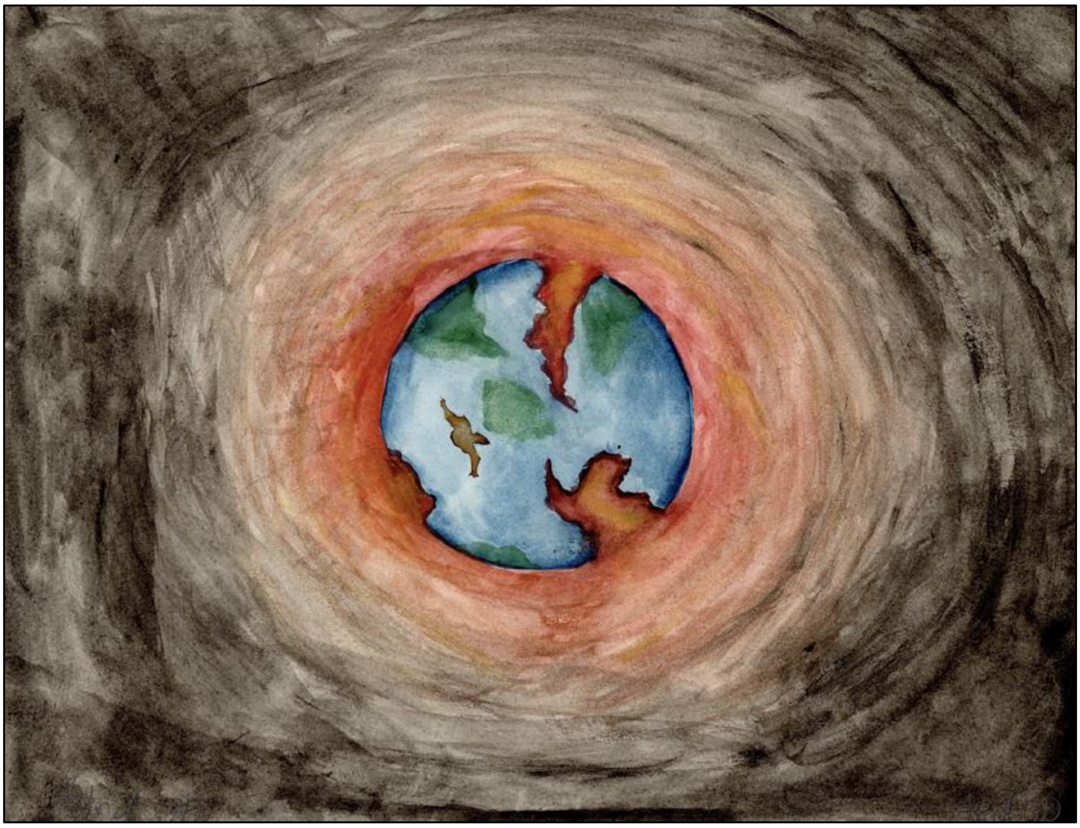

Drawing 41 (no. 416). 13-year-old girl, April 2020.

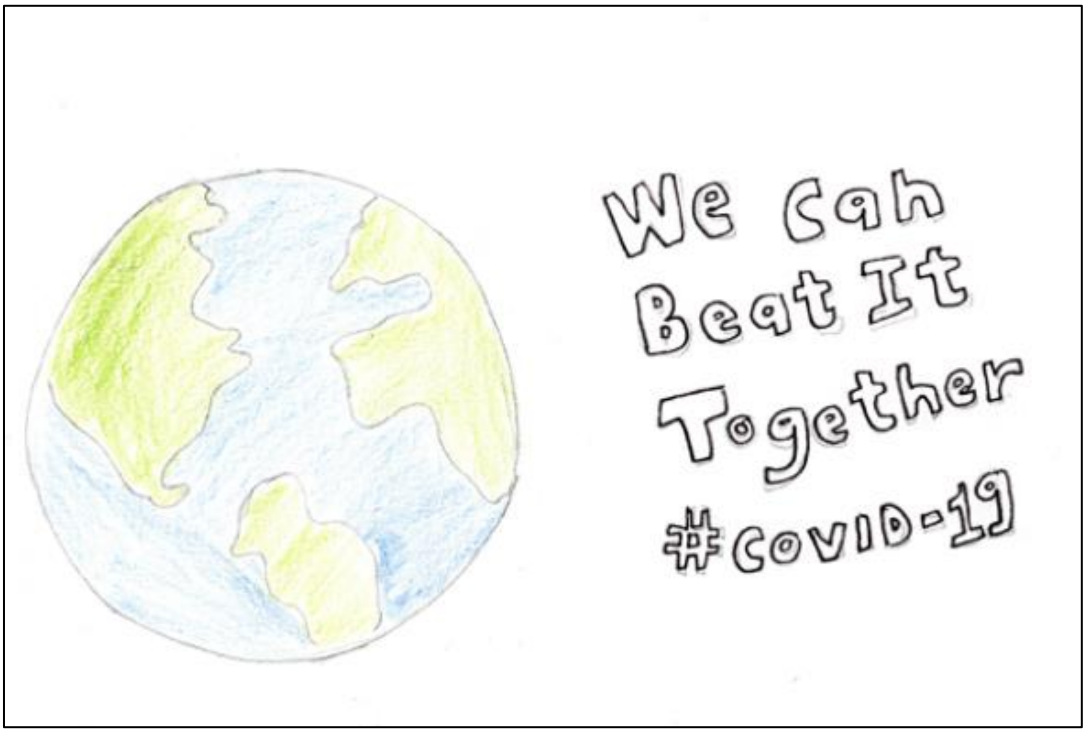

Drawing 42 (no. 717). 15-year-old boy, June 2020.

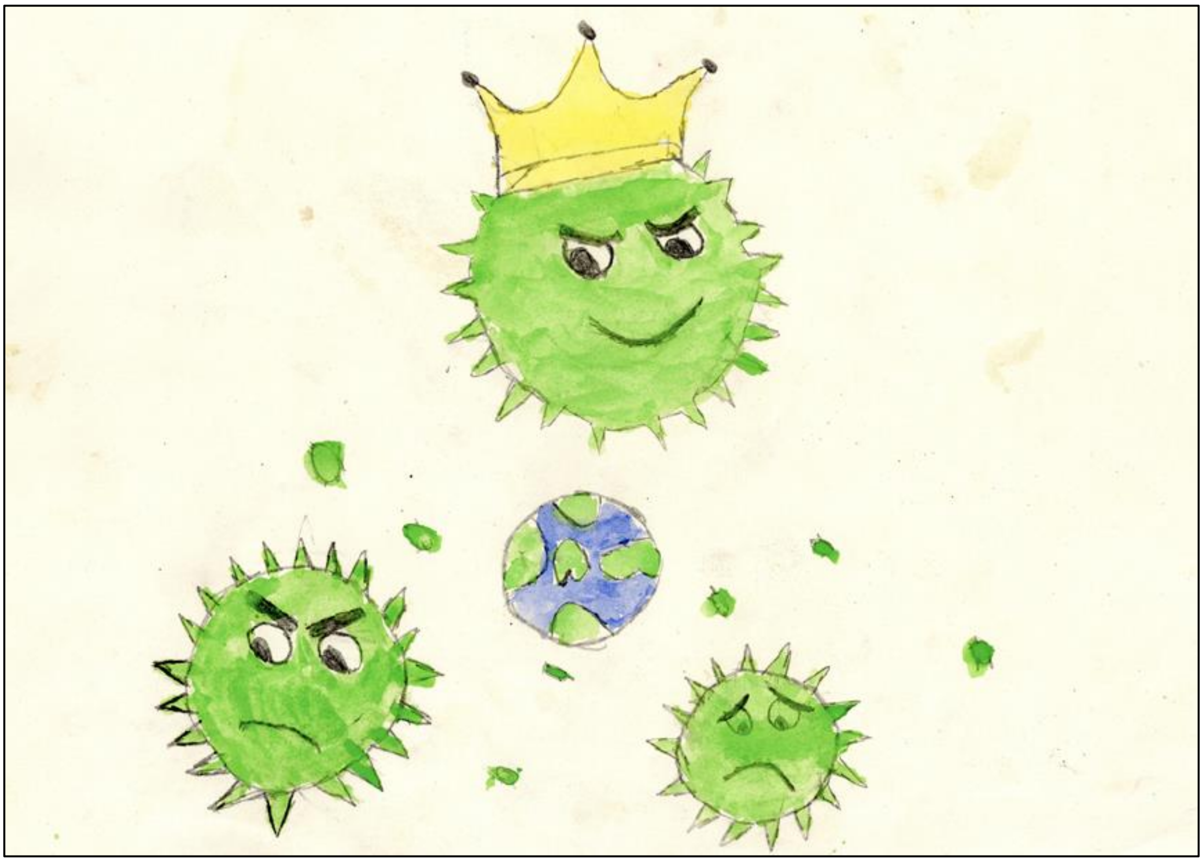

Drawing 43 (no. 427). 13-year-old boy, April 2020.

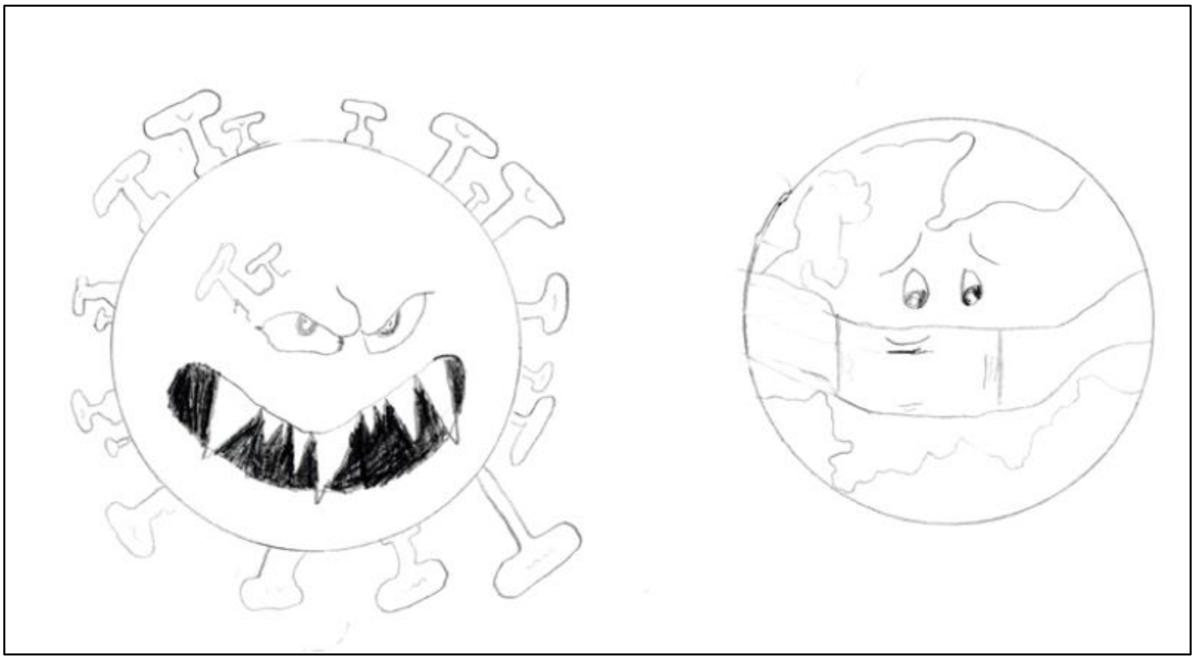

Drawing 44 (no. 655). 13-year-old boy, May 2020.

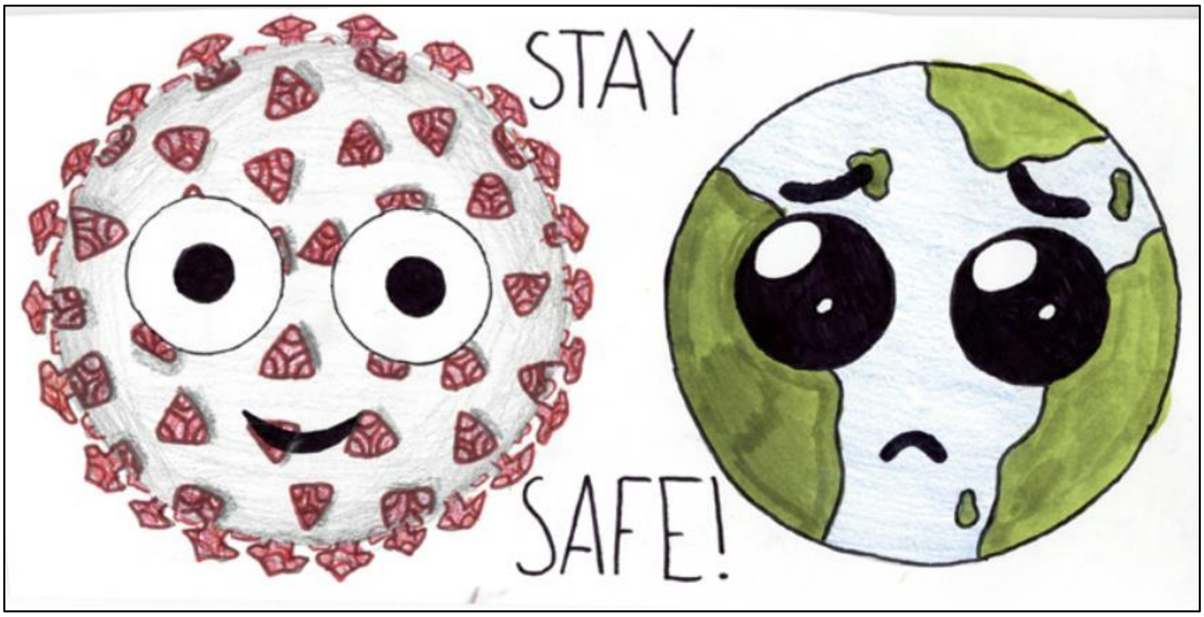

Drawing 45 (no. 773). 14-year-old girl, June 2020.

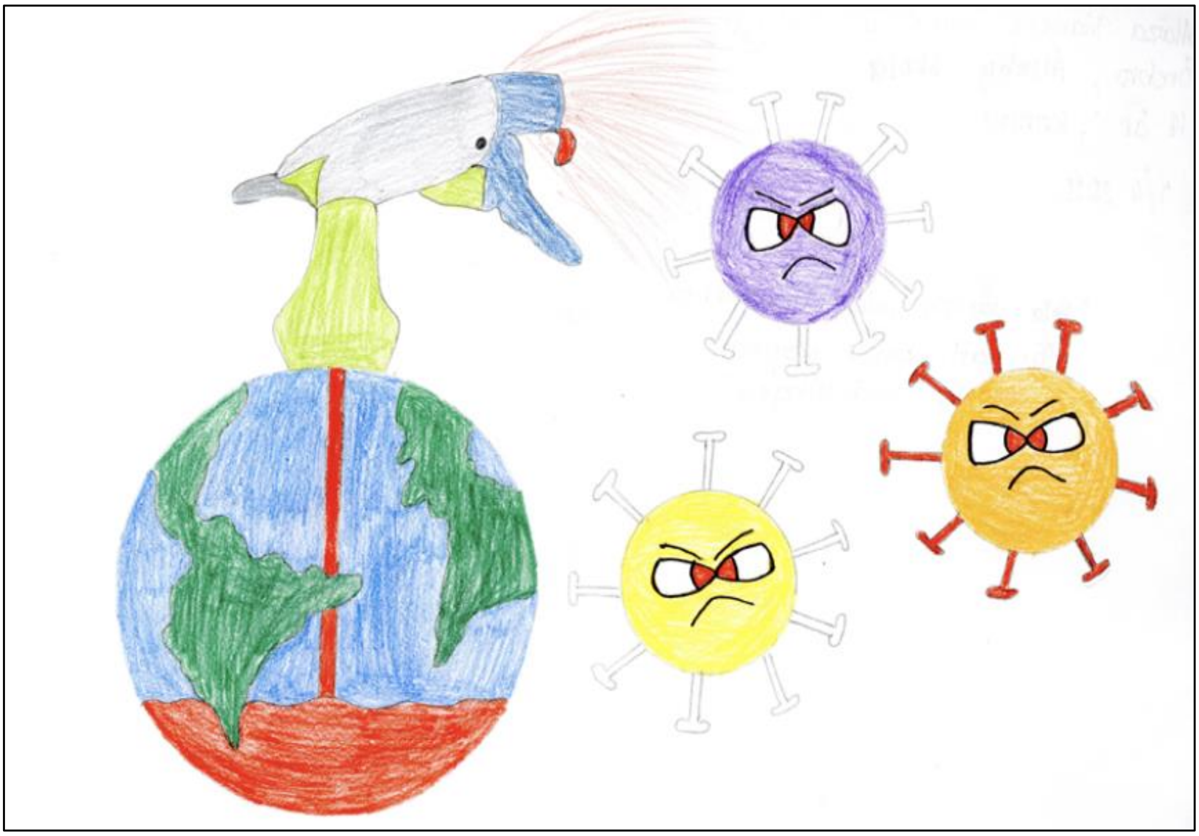

Drawing 46 (no. 727). 14-year-old girl, June 2020.

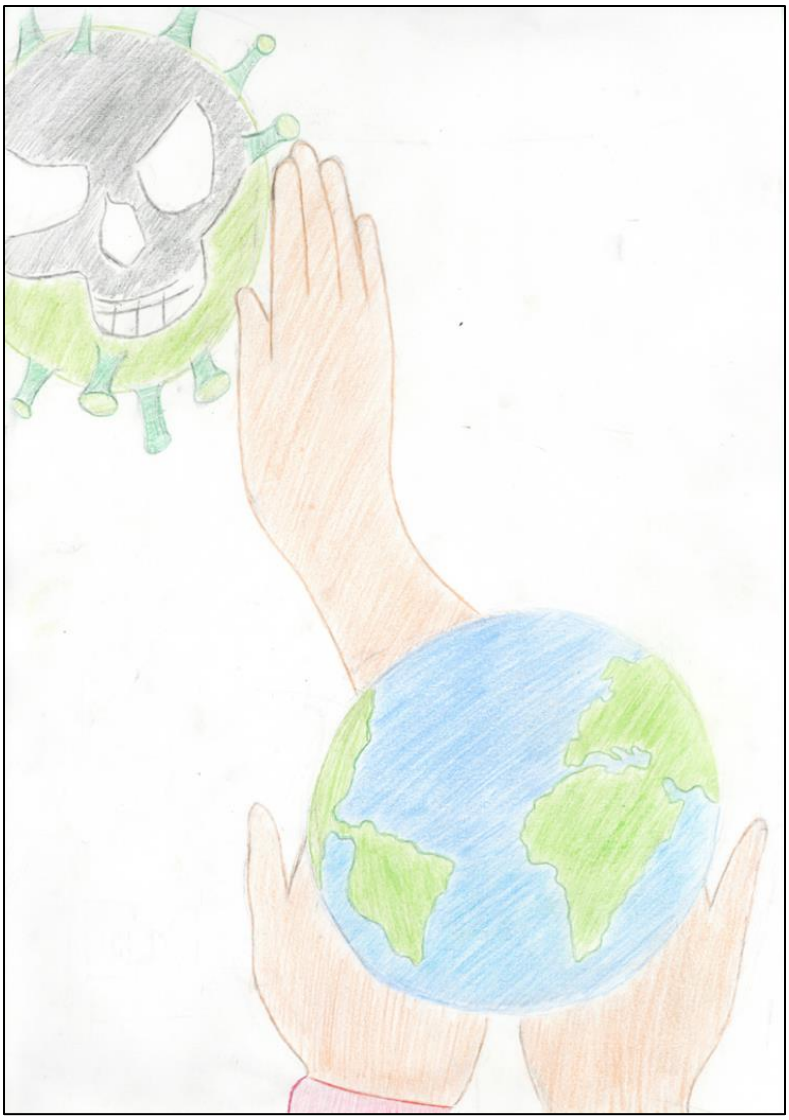

Drawing 47 (no. 900). 13-year-old girl, May 2020.

Motif 6: References to time, or specific times

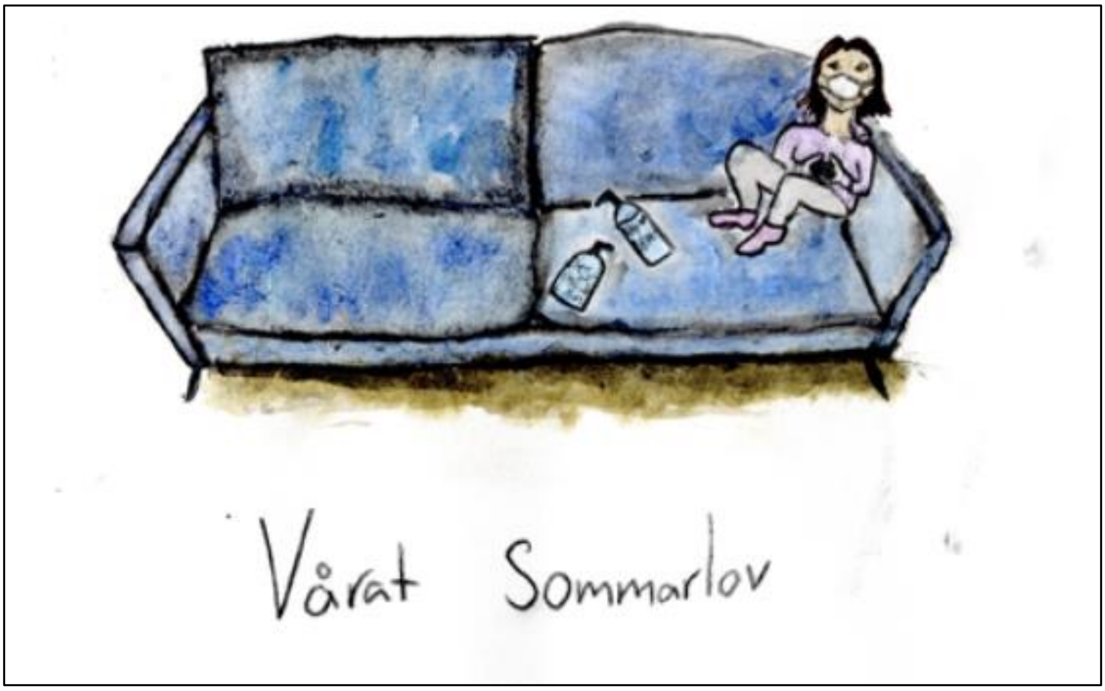

Drawing 48 (no. 211). 13-year-old girl, May 2020.

The title reads "our summer holiday", and the bottles are labelled "hand disinfectant".

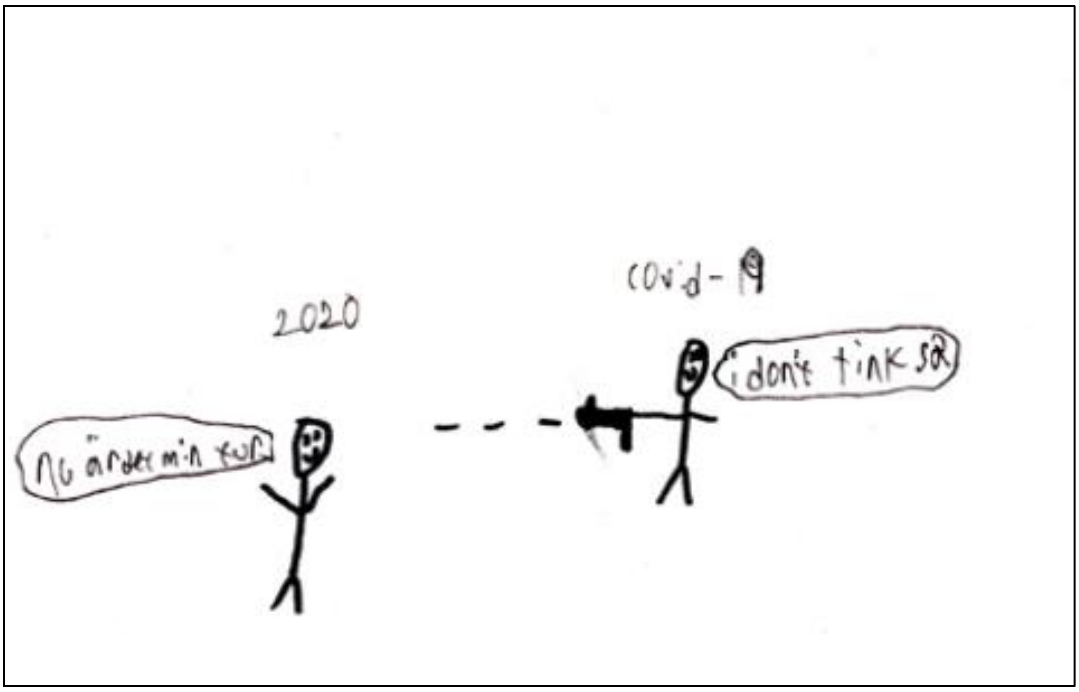

Drawing 49 (no. 666). 14-year-old boy, June 2020.

2020's text reads "now it is my turn", in Swedish. COVID-19 is saying "I don't tink (sic) so", in English.

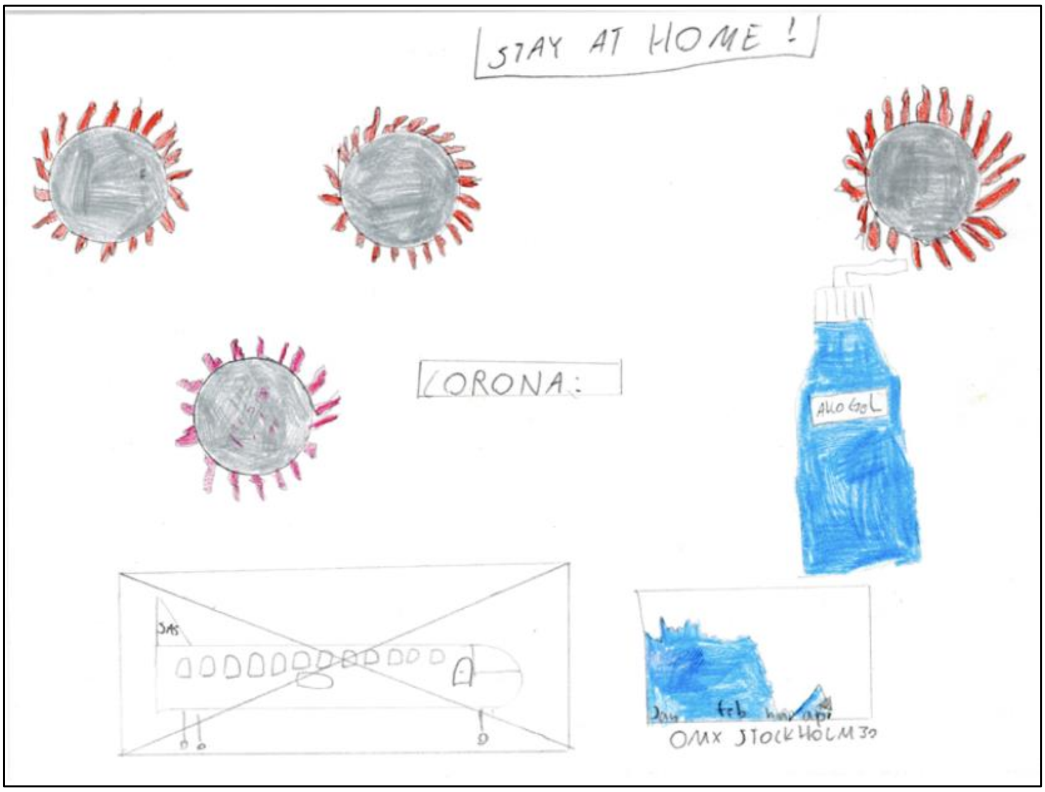

Drawing 50 (no. 700). 14-year-old boy, June 2020.

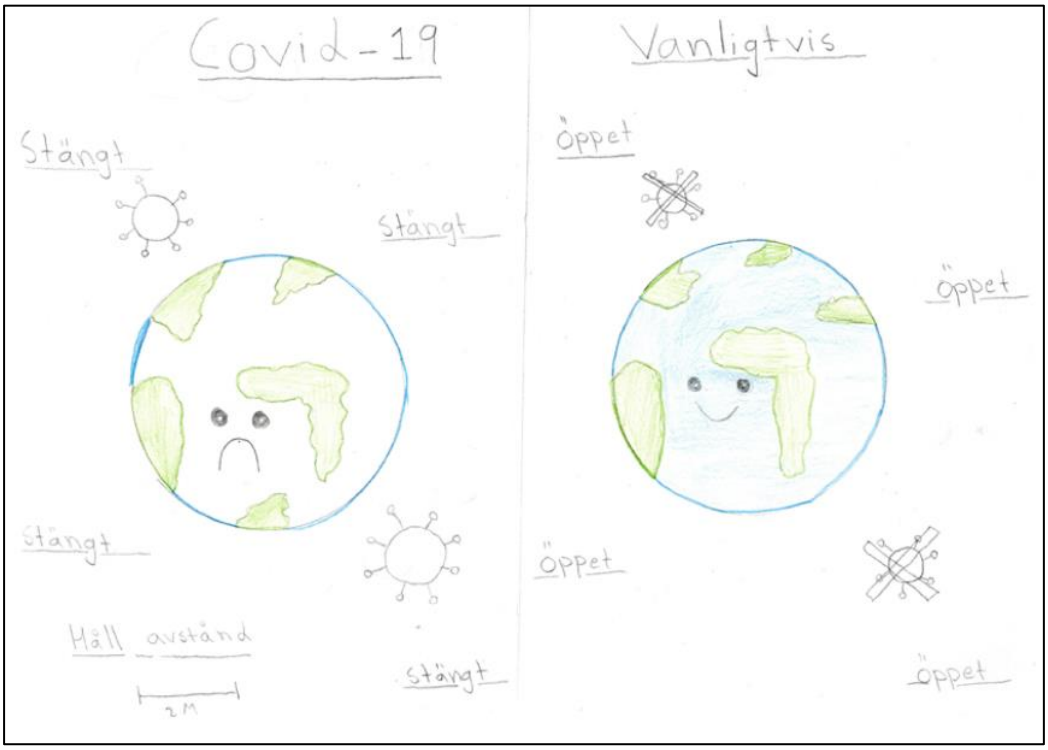

Drawing 51 (no. 669): 14-year-old girl. June 2020.

The titles are “COVID-19” and “usually” respectively, and the text changes from “open” (usually) to “closed” and “keep distance” (COVID-19).

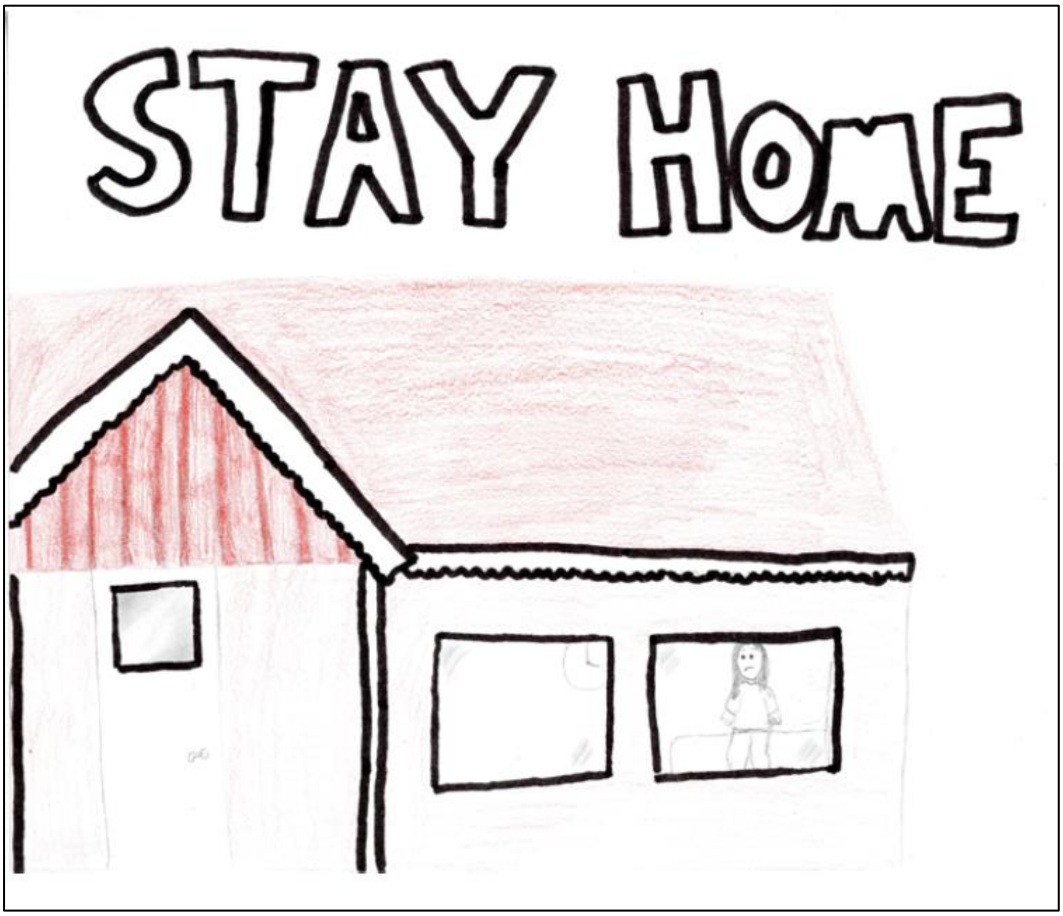

Drawing 52 (no. 714). 14-year-old boy, June 2020.

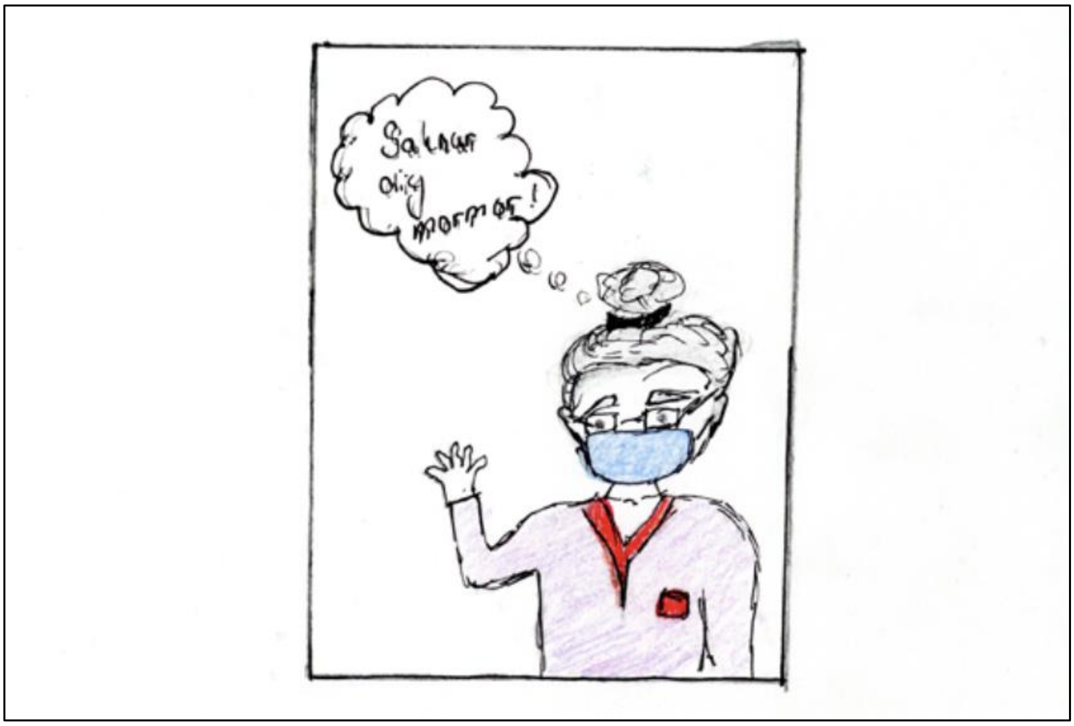

Drawing 53 (no. 770). 14-year-old girl, May 2020.

The text reads: “miss you grandma!”

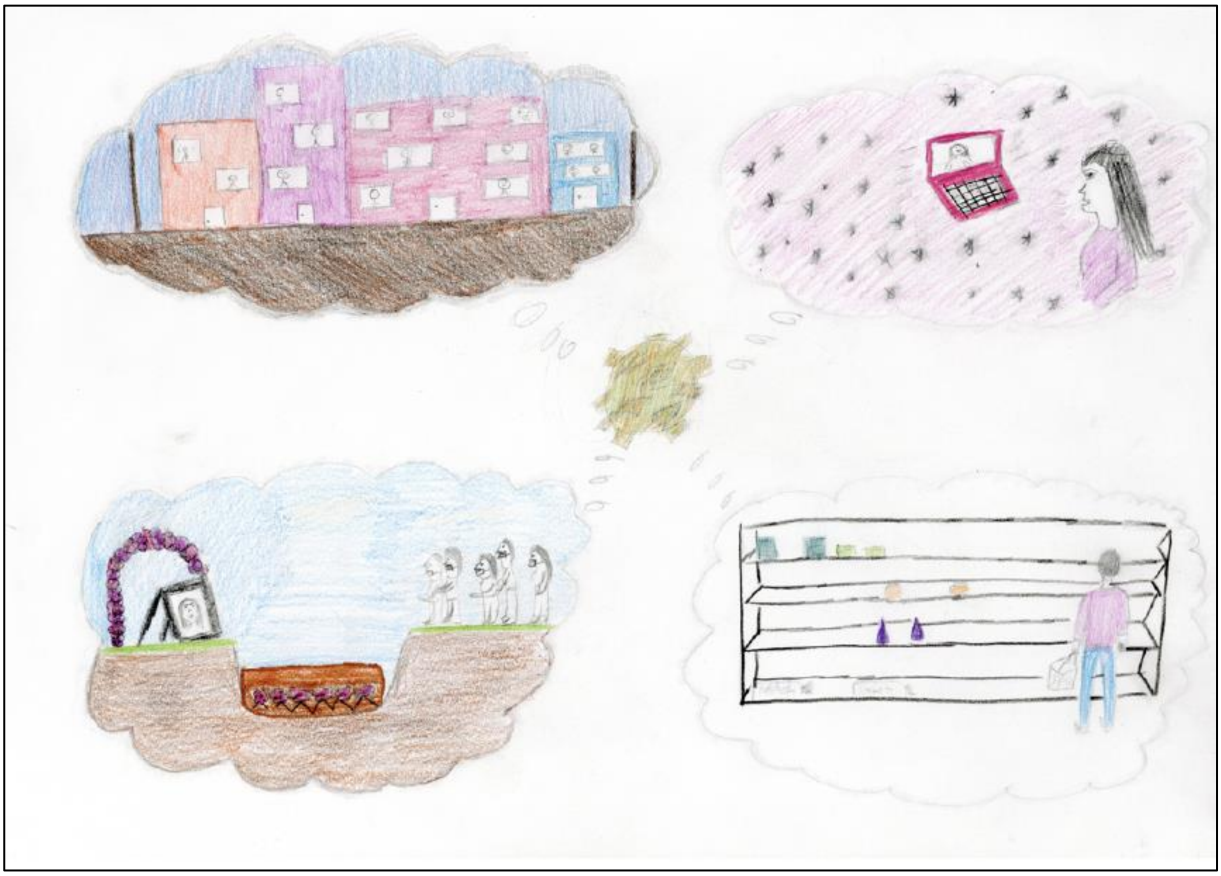

Drawing 54 (no. 889). 14-year-old girl, May 2020.
